# Long-term mortality following robotic mitral valve repair

**DOI:** 10.1016/j.xjse.2025.100090

**Published:** 2025-11-19

**Authors:** Maxwell C. Braasch, Mehran Rahimi, Ahmed Hanafy, Alexandra A. Malarczyk, June He, Mary A. Siki, Takashi Murashita, Harold G. Roberts, Ralph J. Damiano, Tsuyoshi Kaneko

**Affiliations:** Division of Cardiothoracic Surgery, Department of Surgery, Washington University in St. Louis, St. Louis, Mo

**Keywords:** robotic mitral valve, mitral valve repair, degenerative mitral regurgitation, outcomes, long-term mortality

## Abstract

**Objective:**

To compare long-term mortality between robotic and conventional mitral valve repair (MVr) for degenerative mitral regurgitation (DMR).

**Methods:**

A retrospective analysis of Medicare data from 2012-2024 for patients who underwent surgical MVr for DMR was conducted. Patients with prior cardiac surgery were excluded. Two groups were created: robotic MVr and conventional MVr, which included both sternotomy and thoracotomy approaches. The primary outcome was 5-year mortality. Robotic and conventional cases were 1:2 propensity score matched for outcome assessment.

**Results:**

A total of 1502 cases of robotic MVr and 12,182 cases of conventional MVr were identified. The robotic approach represented a growing proportion of surgical MVr. Compared to patients who underwent conventional MVr, patients who underwent robotic MVr were younger and had a lower comorbid disease burden (*P* < .05 for both). Unadjusted mortality was lower following robotic MVr compared to conventional MVr at 30 days (0.93% vs 1.8%; *P* = .013), 1 year (2.4% vs 5.3%; *P* < .001), and 5 years (8.3% vs 15%; *P* < .001). Risk-adjusted mortality was similar in the 2 groups at 30 days (1.2% vs 0.98%; *P* = .48), 1 year (2.8% vs 3.1%; *P* = .72), and 5 years (8.8% vs 9.9%; *P* = .32) to conventional MVr. Hospital length of stay (mean, 6.2 ± 3.9 days vs 8.0 ± 5.0 days; *P* < .001) was shorter and rates of renal failure (6.8% vs 9.6%; *P* = .007) and permanent pacemaker implantation (2.0% vs 4.6%; *P* < .001) were lower following robotic MVr compared to conventional MVr.

**Conclusions:**

Robotic MVr offered comparable long-term survival to conventional MVr in Medicare patients. These results support the use of robotic MVr for DMR for appropriately selected patients and skilled surgeons.

## Graphical Abstract

**Figure:**
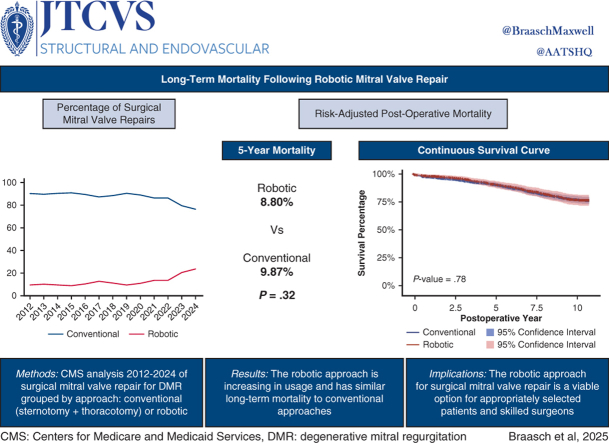

**Figure:**
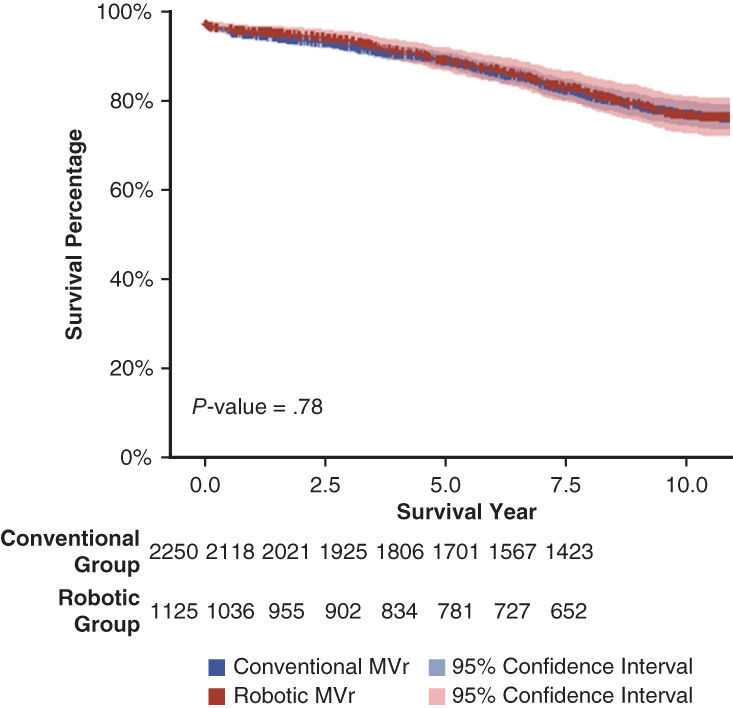
Robotic and conventional mitral valve repair have similar long-term mortality in CMS data. The shaded area represents the 95% confidence interval.

**Central Message**

Robotic and conventional mitral valve repair for degenerative mitral regurgitation have similar long-term mortality in Medicare patients.

**Perspective**

Our analysis showed that both short-term and long-term mortality following robotic mitral valve repair for degenerative mitral regurgitation were comparable to mortality following conventional mitral valve repair. Robotic mitral valve repair is a viable option for appropriately selected patients.

Surgical mitral valve repair (MVr) is the gold standard for treating degenerative mitral regurgitation (DMR) that has shown to improve survival.^1^ As the field of cardiac surgery expanded to minimally invasive approaches from hemi-sternotomy and right mini-thoracotomy, the first robotic MVr was performed by Dr Carpentier in 1996.^2^ The increasing use of the robotic approach for surgical MVr^3^^,^^4^ demands thorough knowledge of its outcomes. Proper patient selection for and outcome analysis of robotic MVr for DMR is critical for improving patient care.

Short-term mortality following robotic MVr has been extensively evaluated and shown to be similar to that associated with sternotomy and thoracotomy approaches.^3^^,^^5^ However, long-term mortality assessment of robotic MVr is limited to single-center analyses.^6^, ^7^, ^8^ Greater understanding of long-term mortality following robotic MVr is needed to better understand the changing landscape of surgical and transcatheter intervention of DMR. The primary objective of this study was to evaluate the long-term mortality following robotic MVr for DMR compared to conventional MVr using a national representative database.

## Methods

### Study Design and Data Sources

**Figure 1:**
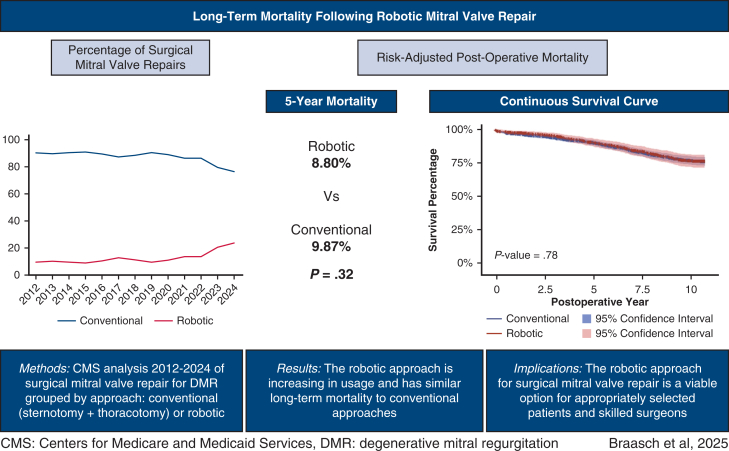
Overall study design and results.

A retrospective design was implemented to evaluate the study objectives (Figure 1). This study was approved by our Institutional Review Board and deemed except from informed consent due to use of deidentified data (IRB #202301098l, approved January 19, 2023). The Centers for Medicare and Medicaid Services (CMS) Virtual Research Data Center database was used to access inpatient for fee-for-service claims for CMS beneficiaries from January 1, 2012 through June 30, 2024. Beneficiary demographic characteristics were collected from the Master Beneficiary Summary File and comorbidity data were collected from the 27 chronic conditions segment.^9^ International Classification of Diseases (ICD) Ninth and Tenth Revision codes were used for procedural, comorbidity, and outcome data collection (Table E1). Hospital data regarding bed size and teaching status was obtained from the 2023 American Hospital Association survey.

### Cohort Identification, Inclusion Criteria, and Exclusion Criteria

**Figure 2:**
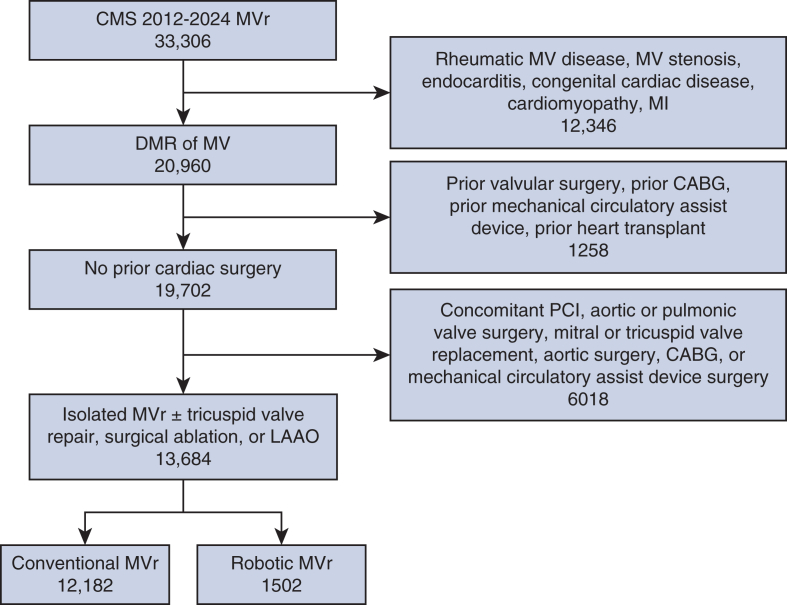
Study cohort selection. *CMS*, Centers for Medicare and Medicaid Services; *MVr*, mitral valve repair; *MV*, mitral valve; *MI*, myocardial infarction; *DMR*, degenerative mitral regurgitation; *CABG*, coronary artery bypass grafting; *PCI*, percutaneous coronary intervention; *LAAO*, left atrial appendage occlusion.

Adult patients were included in analysis if they underwent surgical MVr through ICD-9 and -10 procedure code queries in CMS fee-for-service claims (Figure 2). Patients were excluded if they had a non-DMR etiology of mitral valve disease, including mitral valve stenosis, rheumatic mitral disease, cardiomyopathy, endocarditis, congenital cardiac disease, or myocardial infarction. Patients with a history of prior cardiac surgery were excluded, as were patients undergoing concomitant aortic or pulmonic valve surgery, mitral or tricuspid valve replacement, coronary artery bypass grafting, aortic surgery, mechanical circulatory assist device placement, or percutaneous coronary intervention. The only concomitant cardiac surgeries included in analysis were tricuspid valve repair, surgical ablation, and left atrial appendage occlusion (LAAO). This identified a final cohort of patients without a history of cardiac surgery with DMR who underwent a first-time surgical MVr. ICD-9 and -10 codes were then used to identify patients who underwent a robotic approach for their surgical MVr,^10^ creating the robotic group. Those who did not undergo a robotic approach for their MVr were included in the conventional MVr group, which included patients who underwent MVr via either a sternotomy or a thoracotomy, including a mini-thoracotomy.

### Data Analysis

First, the annual frequencies of robotic MVr and conventional MVr were determined in terms of both the total number of annual surgeries and the annual percentage of total surgeries performed per approach. Baseline patient characteristics for the robotic and conventional groups are reported as frequency with percentage and mean with standard deviation (SD). Comparisons were made across groups using the χ^2^ test for discrete variables and the *t* test for continuous variables. Propensity score matching at a 1:2 ratio was then performed to create equivalent groups for outcome assessment based on the following variables: age, male sex, white race, diabetes mellitus, hypertension, coronary artery disease, atrial fibrillation, congestive heart failure, peripheral vascular disease, chronic obstructive respiratory disease, chronic kidney disease (CKD), hospital bed size, hospital teaching status, concomitant tricuspid valve repair, concomitant surgical ablation, and concomitant LAAO.

Postoperative mortality at 30 days, 1 year, and 5 years were determined for both the unmatched group and propensity score–matched group and compared. The primary outcome was 5-year mortality. Postoperative rates of hospital length of stay, acute respiratory insufficiency, renal failure, blood transfusions, permanent pacemaker implantation (PPMI), cerebrovascular accident, and mitral valve reintervention were determined similarly and compared between the unmatched and propensity score–matched groups. Survival curves for the 2 groups were calculated for overall long-term survival assessment. A multivariable logistic regression model was constructed to evaluate the impact of the robotic MVr approach on 30-day mortality. Statistical significance for all analyses was set at *P* < .05. Finally, a sensitivity analysis was performed to evaluate the association of concomitant tricuspid valve repair with postoperative PPMI in the propensity score–matched robotic MVr cases.

## Results

### Procedural Trends

**Figure 3:**
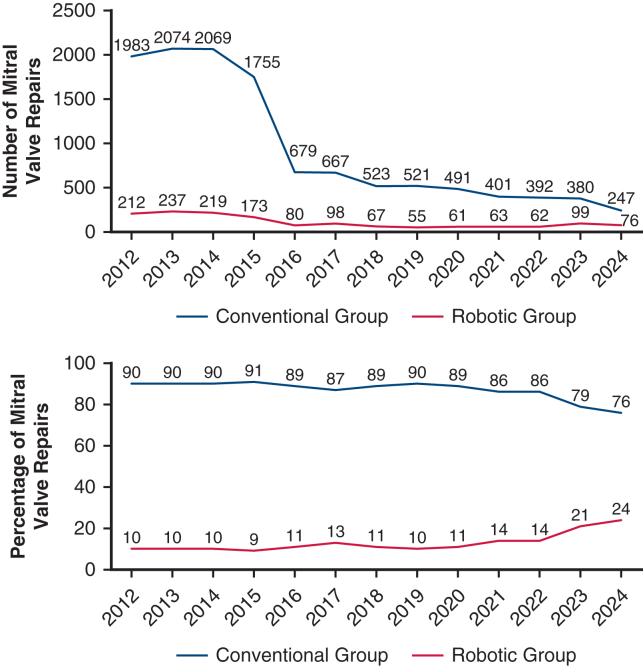
Annual volume of surgical mitral valve repairs for degenerative mitral regurgitation (*top*) and percentage of conventional and robotic mitral valve repair approaches (*bottom*); 2024 with 6 months of data.

A total of 33,306 patients who underwent surgical MVr in the CMS database from January 2012 through June 2024 were identified. After exclusions, the final study cohort comprised 13,684 patients with DMR. Of these, 1502 patients (11%) underwent a robotic MVr and 12,182 (89%) underwent a conventional MVr (Figure 3). The significant drop after 2015 was seen in both groups, likely reflecting the approval of mitral transcatheter edge-to-edge repair (M-TEER). In terms of percentage, between 9% and 14% of all surgical MVr cases were performed via the robotic approach from 2012 through 2022, but this percentage increased to 21% in 2023 and 24% in 2024.

### Patient, Operative, and Hospital Characteristics of Unmatched Groups

Before matching, compared to patients in the conventional MVr group, patients in the robotic MVr group were younger (average age, 71 ± 7 years vs 72 ± 8 years; *P* = .023), were more frequently male (60% vs 55%; *P* < .001), and had a lower burden of all comorbidities assessed (*P* < .05 for all), with the exception of peripheral vascular disease (7.7% vs 7.5%; *P* = .73) (Table 1). Patients in the robotic MVr group underwent fewer concomitant tricuspid valve repairs (3.3% vs 8.7%; *P* < .001) and surgical ablations (5.1% vs 12%; *P* < .001), but more concomitant LAAO procedures (42% vs 35%; *P* < .001). Compared to hospitals in which conventional MVr was performed, hospitals in which robotic MVr was performed were larger and more frequently teaching hospitals (*P* < .001 for both).

**Table 1:** Baseline patient and hospital variables before and after propensity score matching

Variable	Before matching			After matching		
	Robotic MVr group (N = 1502)	Conventional MVr group (N = 12,182)	*P* value	Robotic MVr group (N = 1125)	Conventional MVr group (N = 2250)	*P* value
Age, y, mean ± SD	71 ± 7	72 ± 8	.023	71 ± 8	71 ± 8	.43
Female sex, n (%)	903 (60)	6688 (55)	<.001	668 (59)	1336 (60)	.80
Race, n (%)			<.004			.96
White	1349 (90)	10,927 (90)		1007 (90)	2029 (90)	
Black or African American	56 (3.7)	557 (4.6)		50 (4.4)	92 (4.1)	
Asian	17 (1.1)	161 (1.3)		17 (1.5)	33 (1.5)	
Other	53 (3.3)	322 (2)		47 (4.2)	97 (4.3)	
Diabetes mellitus, n (%)	153 (10)	1791 (15)	<.001	115 (10)	247 (11)	.50
Hypertension, n (%)	978 (65)	8613 (71)	<.001	726 (65)	1422 (63)	.45
Hyperlipidemia, n (%)	731 (49)	6298 (52)	.026	543 (48)	1125 (50)	.34
Chronic kidney disease, n (%)	117 (7.8)	1839 (15)	<.001	83 (7.4)	163 (7.2)	.96
COPD	297 (20)	3594 (30)	<.001	229 (20)	435 (19)	.48
Coronary artery disease	457 (30)	4580 (38)	<.001	356 (32)	681 (30)	.41
Cerebrovascular disease	67 (4.3)	713 (5.9)	.016	52 (4.6)	85 (3.8)	.24
Congestive heart failure	562 (37)	5202 (43)	<.001	458 (41)	869 (39)	.24
Atrial fibrillation	652 (43)	6834 (56)	<.001	592 (53)	1195 (3)	.72
Peripheral vascular disease	116 (7.7)	911 (7.5)	.073	93 (8.3)	186 (8.3)	>.99
Prior myocardial infarction	32 (2.1)	549 (4.5)	<.001	23 (2)	54 (2)	.51
Hospital bed size			<.001			.10
<100 beds	21 (1.4)	296 (2.3)		16 (1.4)	29 (1.3)	
100-400 beds	391 (26)	3729 (31)		293 (26)	586 (26)	
>400 beds	1089 (73)	8172 (67)		816 (73)	1509 (67)	
Teaching hospital	1450 (96)	10,492 (86)	<.001	1086 (97)	2176 (97)	.79
Concomitant procedures						
LAAO	635 (42)	4203 (35)	<.001	422 (38)	826 (37)	.65
Surgical ablation	76 (5.1)	1437 (12)	<.001	50 (4.4)	106 (4.7)	.73
Tricuspid valve repair	50 (3.3)	1062 (8.7)	<.001	38 (3.4)	79 (3.5)	.84

*MVr*, Mitral valve repair; *SD*, standard deviation; *COPD*, chronic obstructive pulmonary disease; *LAAO*, left atrial appendage occlusion.

### Patient, Operative, and Hospital Characteristics of Propensity Score–Matched Groups

After 1:2 propensity score matching, there were 1125 patients in the robotic MVr group and 2250 patients in the conventional MVr group (Table 1). Patients in the 2 groups were of similar average age (71 ± 8 years vs 71 ± 8 years; *P* = .43), had a similar male preponderance (59% vs 60%; *P* = .80), and had a similar comorbid disease burden (*P* > .05 for all). In addition, rates of concomitant tricuspid valve repairs (3.4% vs 3.5%; *P* = .84), surgical ablation (4.4% vs 4.7%; *P* = .73), and LAAO (38% vs 37%; *P* = .65) were similar in the 2 groups. Hospitals in the 2 groups were similar with respect to bed size and teaching status (*P* > .05 for both).

### Postoperative Mortality and In-Hospital Outcomes of the Unmatched Groups

**Figure E1:**
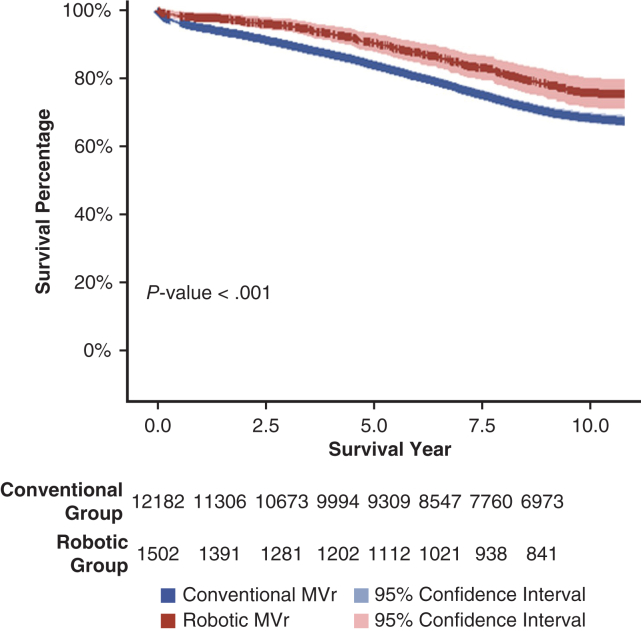
Unadjusted survival curves following conventional and robotic mitral valve repair (*MVr*).

Mortality following MVr was lower at 30 days (0.93% vs 1.8%; *P* = .013), 1 year (2.4% vs 5.3%; *P* < .001), and 5 years (8.3% vs 15%; *P* < .001) in the robotic MVr group compared to the conventional MVr group (Table E2). Mitral valve reintervention was similar in the robotic and conventional groups (4.6% vs 4.5%; *P* = .90). Average hospital length of stay was shorter in the robotic MVr group (6.3 ± 3.8 days vs 8.8 ± 5.9 days; *P* < .001). Kaplan-Meier survival analysis demonstrated superior survival for the robotic MVr group compared to the conventional MVr group (*P* < .001; Figure E1).

### Postoperative Mortality and In-Hospital Outcomes of the Propensity Score–Matched Groups

**Figure 4:**
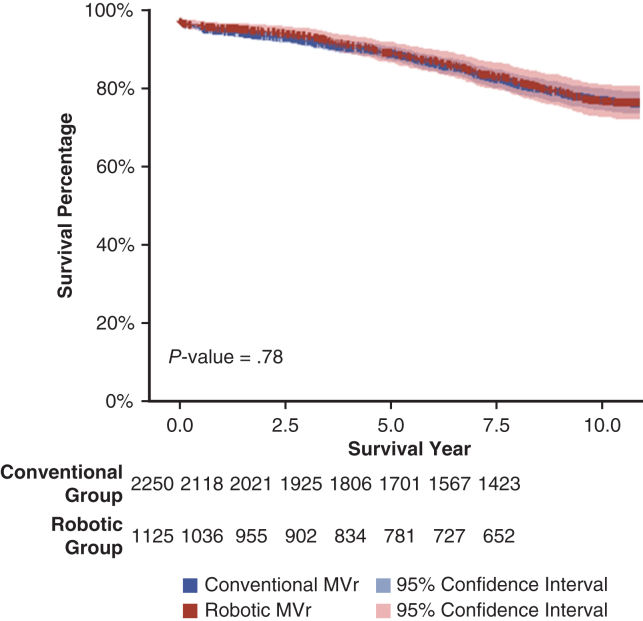
Risk-adjusted survival curves following conventional mitral valve repair (*MVr*) and robotic MVr.

Following propensity score matching, mortality following MVr was similar in the robotic group and the conventional group at 30 days (1.2% vs 0.98%; *P* = .48), 1 year (2.8% vs 3.1%; *P* = .72), and 5 years (8.8% vs 9.9%; *P* = .32) (Table 2). Mitral valve reintervention also was in the 2 groups (4.6% vs 4%; *P* = .51). Patients in the robotic MVr group had a shorter average hospital length of stay (6.2 ± 3.9 days vs 8.0 ± 5.0 days; *P* < .001) and lower rates of renal failure (6.8% vs 9.6%; *P* = .007) and PPMI (2.0% vs 4.6%; *P* < .001). Risk-adjusted survival curves demonstrated similar survival in the robotic MVr and conventional MVr groups across the full 12.5-year study period (*P* = .78; Figure 4). On sensitivity analysis of robotic MVr, concomitant tricuspid valve repair was not associated with PPMI compared to no concomitant tricuspid valve repair (0% vs 2.1%; *P* = .37) (Table E3).

**Table 2:** Postoperative morbidity and mortality following MVr for propensity score–matched groups

Parameter	Robotic MVr group (N = 1125)	Conventional MVr group (N = 2250)	*P* value
Hospital length of stay, d, mean ± SD	6.2 ± 3.9	8 ± 5	<.001
Acute pulmonary insufficiency	45 (4)	101 (4.5)	.51
Acute renal failure	77 (6.8)	216 (9.6)	.007
Blood product transfusion	212 (19)	441 (20)	.60
PPMI	23 (2)	104 (4.6)	<.001
Cerebrovascular accident	11 (1)	12 (1)	.14
Mitral valve reintervention	52 (4.6)	91 (4)	.51
Mortality			
30 d	14 (1.2)	22 (0.98)	.48
1 y	32 (2.8)	69 (3.1)	.72
5 y	99 (8.8)	222 (9.9)	.32

*MVr*, Mitral valve repair; *SD*, standard deviation; *PPMI*, permanent pacemaker implantation.

### Multivariable Analysis of Post-operative Mortality

**Figure 5:**
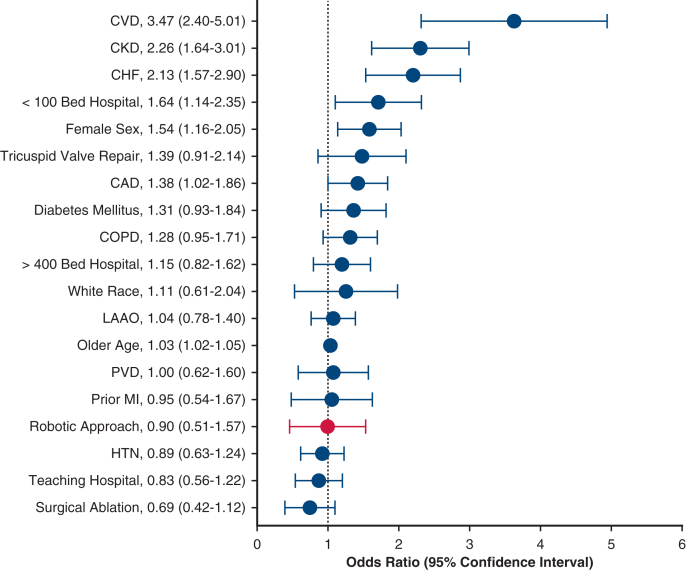
Multivariable logistic regression of factors associated with 30-day mortality following surgical mitral valve repair. *CVD*, Cerebrovascular disease; *CKD*, chronic kidney disease; *CHF*, congestive heart failure; *CAD*, coronary artery disease; *COPD*, chronic obstructive respiratory disease; *LAAO*, left atrial appendage occlusion; *PVD*, peripheral vascular disease; *MI*, myocardial infarction; *HTN*, hypertension.

Multivariable analysis demonstrated no association between a robotic approach to MVr and 30-day mortality (odds ratio [OR], 0.90; 95% confidence interval [CI] 0.51-1.57) (Figure 5). Variables associated with increased risk of postoperative mortality following MVr included prior cerebrovascular disease (OR, 3.47; 95% CI, 2.40-5.01), CKD (OR, 2.26; 95% CI, 1.64-3.01), congestive heart failure (OR, 2.13; 95% CI 1.57-2.90), <100-bed hospital (OR, 1.64; 95% CI, 1.14-2.35), female sex (OR, 1.54; 95% CI, 1.16-2.05), coronary artery disease (OR, 1.38; 95% CI, 1.02-1.86), and increasing age (OR, 1.03; 95% CI, 1.02-1.05).

## Discussion

This study comparing the long-term mortality following robotic MVr with that after conventional MVr in a CMS population yielded several important findings. First, the robotic approach represents an increasing proportion of the total surgical MVr cases in this population. Second, long-term mortality following robotic MVr is comparable to that after conventional MVr. Third, hospital length of stay and rates of PPMI and renal failure were lower following robotic MVr than after conventional MVr. Finally, multivariable analysis demonstrated that the robotic approach was not associated with increased post-MVr mortality. These results demonstrate that the robotic approach performed by a skilled surgeon is a viable option for MVr in appropriately selected patients.

Robotic MVr is an increasingly popular approach in surgical MVr. The growing proportion of surgical MVr being performed robotically relative to conventional approaches has been demonstrated in 2 recent analyses of the Society of Thoracic Surgeons (STS) database.^3^^,^^11^ While our study population was an older cohort of CMS patients, we also found an increasing proportion of surgical MVr performed robotically for DMR, with an increase from 14% in 2022 to 24% in 2024. This increase is particularly significant given the growth of M-TEER.^12^ The decline in total annual surgical MVr cases observed in our results and in prior analysis^12^ is likely secondary to the approval of M-TEER in 2013.^13^ Interestingly, surgical MV replacement continues to represent approximately 40% of all surgical mitral interventions among CMS patients with DMR from 2012-2019.^12^ With the ongoing clinical evaluation of M-TEER versus surgery in patients with DMR,^14^^,^^15^ the importance of choosing the proper procedure for each patient, whether transcatheter or surgical MVr, cannot be understated. Continued research to expand the knowledge base of robotic MVr use and outcomes will provide guidance in the changing landscape of intervention for mitral regurgitation.

Short-, intermediate-, and long-term mortality were comparable following robotic MVr and conventional MVr in our CMS analysis, consistent with previous single-institution^6^, ^7^, ^8^ and nationwide analyses.^3^^,^^5^ Multivariable analysis demonstrated that the robotic approach for MVr did not impact postoperative mortality compared to conventional approaches. An important consideration in our results is the combination of sternotomy and thoracotomy approaches in a single group. It was not possible to separate these approaches owing to limitations of the database. A recent study of the STS database demonstrated similar short-term mortality of robotic MVr performed via sternotomy and thoracotomy approaches.^3^ Long-term mortality in the robotic and sternotomy approach for MVr also appeared to be similar in a recently-published single-center study of adults age ≥65 years with DMR.^8^ Given the comparable results between approaches in these prior analyses, this could be true for long-term mortality as well. Our results also add to the existing evidence indicating that robotic mitral valve surgery is safe in older adults.^5^^,^^8^^,^^16^ While single-center analyses have reported 96% to 97% 5-year survival following robotic MVr,^6^, ^7^, ^8^ we observed a 5-year survival following robotic MVr of 92% in our unmatched group and 91% in our propensity score–matched group. This difference is likely due to younger patients in these single-center analyses compared to our CMS patient cohort. The rate of reintervention following robotic MVr was comparable to that following conventional MVr, consistent with prior analysis.^8^ While this does not directly reflect recurrent mitral regurgitation, this result supports the durability of the robotic approach for MVr.

Our results demonstrate short-term benefits of robotic MVr, including shorter hospital length of stay and lower rates of renal failure and PPMI. These are important benefits of the robotic approach that reduce the burden on a limited-resource healthcare system compared to conventional MVr approaches. Prior analyses have shown that robotic MVr is associated with shorter hospital length of stay compared to conventional MVr, even when thoracotomy and sternotomy are analyzed separately.^3^^,^^5^ Robust risk adjustment for preoperative atrial fibrillation and CKD, along with concomitant surgical ablation and LAAO at the time of MVr, reduce the possible impact of procedural selection bias on lower rates of renal failure and PPMI following robotic MVr compared to conventional MVr. Nevertheless, more granular analysis is needed to provide an explanation for these interesting findings, given the limitations inherent to using a claims-based database without operative details.

The increased rate of LAAO in the robotic group compared to the conventional group before matching may be attributed to technical considerations with the robotic approach,^3^ possibly related to improved exposure of the left atrial appendage with the robotic procedure. Only 12% of our conventional MVr patients and 5.1% of robotic MVr patients underwent surgical ablation, despite preoperative atrial fibrillation rates of 56% in the conventional group and 43% in the robotic group. While nationwide rates of surgical ablation with mitral valve surgery are steadily increasing,^11^ our results demonstrate the ongoing need for improvements in the use of surgical ablation, especially for the robotic approach, given the class I indication for surgical ablation in patients with a history of atrial fibrillation undergoing nonemergent mitral valve surgery.^4^

While there is growing utilization of and enthusiasm for the robotic approach to mitral valve surgery,^3^^,^^4^ the selection of robotic procedures must be done carefully to ensure optimal outcomes. A thorough methodology for patient evaluation and selection for the robotic approach is needed, including anatomic evaluation of the chest and consideration of postoperative quality of life benefits of the robotic approach.^17^, ^18^, ^19^ Similar to overall mitral valve surgery,^20^ higher hospital volume of robotic MVr is associated with improved patient outcomes,^3^ and the learning curve for new operators may be as long as 2 years.^6^ Effective robotic operators must be facile with open MVr, as this technique is highly transferrable from the conventional to the robotic approach. Intentional training in conventional and robotic approaches, both during formal cardiac surgical training programs and during independent practice, is necessary to reduce the learning curve and add a high-quality robotic approach for MVr into the armamentarium of more cardiac surgeons.

### Limitations

Some limitations of this study must be acknowledged. We were unable to differentiate MVr performed via sternotomy or via thoracotomy owing to limitations in ICD coding; however, given that prior analyses have shown similar short-term mortality with robotic MVr and conventional MVr via sternotomy or thoracotomy,^3^ this limitation might not have had a significant impact on the primary outcome. As this is a retrospective study, there is inherent selection bias between robotic MVr and conventional MVr that cannot be fully eliminated. Rigorous analysis through propensity score matching and multivariable logistic regression were used to minimize the influence of procedural selection bias. The results of this study of CMS beneficiaries might not be generalized to a younger population. It is possible that patients with ischemic mitral regurgitation could have been included in this analysis of patients with DMR given the limitations of utilization of a claims-based registry; however, our use of robust methodology^12^ for removing patients with non-DMR etiology minimized this risk. Finally, CMS data do not include any imaging or laboratory data or data on disease-specific mortality, precluding a more granular analysis, including recurrent mitral regurgitation. Our primary outcome—5-year mortality—is not impacted by this limitation.

## Conclusions

Robotic MVr is accounting for an increasing proportion of surgical MVr performed in Medicare beneficiaries, particularly in recent years. Short-, intermediate-, and long-term mortality following robotic MVr is comparable to that with conventional MVr approaches. The robotic approach to MVr performed by skilled surgeons should continue to be offered to appropriately selected patients.

## Conflict of Interest Statement

Dr Damiano reports serving as a consultant for Medtronic and Edwards Lifesciences and as a speaker for AtriCure and receiving research funding from AtriCure. Dr Kaneko discloses his relationship as a consultant for Medtronic and an advisory board member for Edwards Lifesciences, Abbott, Anteris, and 4C Medical. All other authors reported no conflicts of interest.

The *Journal* policy requires editors and reviewers to disclose conflicts of interest and to decline handling or reviewing manuscripts for which they may have a conflict of interest. The editors and reviewers of this article have no conflicts of interest.

## Figures and Tables

**Table E1:** ICD-9 and ICD-10 codes used for data collection

Inclusion criteria	
Surgical MVr	ICD-9: 3502, 3512 ICD-10: 02QG0ZE, 02QG0ZZ, 02QG3ZE, 02QG3ZZ, 02QG4ZE, 02QG4ZZ
Robotic system used	ICD-9: 17.41, 17.42, 17.43, 17.44, 17.45, 17.49 ICD-10: 8E0W0CZ, 8E0W0EN, 8E0W0EZ, 8E0W3CZ, 8E0W3EN, 8E0W3EZ, 8E0W4CZ, 8E0W4EN, 8E0W4EZ, 8E0W7CZ, 8E0W7EN, 8E0W7EZ, 8E0W8CZ, 8E0W8EN, 8E0W8EZ, 8E0WXBF, 8E0WXBG, 8E0WXBH, 8E0WXBZ, 8E0WXCZ, 8E0WXY8
Exclusion criteria	
Rheumatic mitral valve disease or mitral valve stenosis	ICD-9: 394, 3940, 3941, 3942, 3949, 396, 3960, 3961, 3962, 3963, 3968, 3969 ICD-10: I080, I081, I083, I088, I089, I050, I051, I059, I052, I058, I342
Endocarditis	ICD-9: 421, 4210, 4211, 4219, 4249, 42490, 42491, 42499, 11281, 07422, 4148, 4149 ICD-10: I011, A3282, A3951, A5203, A5483, B3321, B376, I33, I330, I339, I38, I39, M3211
Congenital cardiac disease	ICD-9: 7450, 7451, 74510, 74511, 74512, 74519, 7452, 7453, 7454, 74560, 74561, 74569, 7457, 7458, 7462, 7465, 7466, 7467 ICD-10: Q210, Q221, Q231, Q244, Q2543, Q263, Q87418, Q20, Q200, Q201, Q202, Q203, Q204, Q205, Q206, Q208, Q209, Q213, Q212, Q218, Q219, Q225, Q232, Q233, Q234, Q225
Cardiomyopathy	ICD-9: 425, 4250, 4251, 42511, 42518, 4252, 4253, 4254, 4255, 4257, 4258, 4259 ICD-10: I255, I421, I420, I422, I427, I428, I429, I42, I421, I423, I424, I425, I426, I43
Myocardial infarction	ICD-9: 410, 4100, 41001, 41002, 41003, 4101, 41010, 41011, 41012, 4102, 41020, 41021, 41022, 4103, 41030, 41031, 41032, 4104, 41040, 41041, 41042, 4105, 41050, 41051, 41052, 4106, 41060, 41061, 410, 4100, 41001, 41002, 41003, 4101, 41010, 41011, 41012, 4102, 41020, 41021, 41022, 4103, 41030, 41031, 41032, 4104, 41040, 41041, 41042, 4105, 41050, 41051, 41052, 4106, 41060, 41061, 41062, 4107, 41070, 41071, 41072, 4108, 41080, 41081, 41081, 4109, 41090, 41091, 41092, 41062, 4107, 41070, 41071, 41072, 4108, 41080, 41081, 41081, 4109, 41090, 41091, 41092 ICD-10: All I21, I22, I23 codes (I210, I2101, I2102, I211, I2111, I2119, I212, I2121, I2129, I213, I214, I219, I213, I213, I214, I21A, I21A1, I219), I252, all I257
Prior CABG	ICD-9: V45.81 ICD-10: Z95.1
Prior valve replacement	ICD-9: V43.3, V42.2 ICD-10: Z95.2, Z95.3, Z95.4
Prior heart assist device	ICD-9: V43.21 ICD-10: Z95.811
Prior heart transplant	ICD-9: 3751 ICD-10: Z94.1
Aortic valve surgery	ICD-9: 3521, 3522, 3511, 3501, 3505, 3506, 3509, 3510, 3511, 3533, 3595, 3596, 3599 ICD-10: 02RF07Z, 02RF08Z, 02RF0JZ, 02RF0KZ, 02RF37H, 02RF37Z, 02RF38H, 02RF38Z, 02RF3JH, 02RF3JZ, 02RF3KH, 02RF3KZ, 02RF47Z, 02RF48Z, 02RF4JZ, 02RF4KZ, 02QF0ZZ, 02QF3ZZ, 02QF4ZZ, 027F0ZZ, 027F3ZZ, 027F4ZZ, 027F04Z, 027F34Z, 027F44Z, 027F0DZ, 027F3DZ, 027F4DZ, 02NF0ZZ, 02NF3ZZ, 02NF4ZZ, 02UF07Z, 02UF0JZ, 02UF0KZ, 02UF37Z, 02UF3JZ, 02UF3KZ, 02UF47Z, 02UF4JZ, 02UF4KZ, 02BF0ZZ, 02BF3ZZ, 02BF4ZZ, 025F0ZZ, 025F3ZZ, 025F4ZZ, X2RF032, X2RF432, X2RF332, 027F34Z, 027F44Z, 02QF0ZJ, 02QF3ZJ, 02QF4ZJ, 02WF07Z, 02WF08Z, 02WF0JZ, 02WF0KZ, 02WF37Z, 02WF38Z, 02WF3JZ, 02WF3KZ, 02WF47Z, 02WF48Z, 02WF4JZ, 02WF4KZ, 025F0ZZ, 025F3ZZ, 025F4ZZ, 027F04Z, 027F0DZ, 027F0ZZ, 027F3DZ, 027F3ZZ, 027F4DZ, 027F4ZZ, 02BF0ZZ, 02BF3ZZ, 02BF4ZZ, 02CF0ZZ, 02CF3ZZ, 02CF4ZZ, 02NF0ZZ, 02NF3ZZ, 02NF4ZZ, 02QF0ZZ, 02QF3ZZ, 02QF4ZZ
Pulmonic valve surgery	ICD-9: 3525, 3526, 3513, 3503, 3507, 3508 ICD-10: 02RH07Z, 02RH08Z, 02RH0JZ, 02RH0KZ, 02RH37H, 02RH37Z, 02RH38H, 02RH38Z, 02RH3JH, 02RH3JZ, 02RH3KH, 02RH3KZ, 02RH47Z, 02RH48Z, 02RH4JZ, 02RH4KZ, 02QH0ZZ, 02QH3ZZ, 02QH4ZZ, 027H0ZZ, 027H3ZZ, 027H4ZZ, 027H04Z, 027H34Z, 027H44Z, 027H0DZ, 027H3DZ, 027H4DZ, 02NH0ZZ, 02NH3ZZ, 02NH4ZZ, 02UH07Z, 02UH0JZ, 02UH0KZ, 02UH37Z, 02UH3JZ, 02UH3KZ, 02UH47Z, 02UH4JZ, 02UH4KZ, 02BH0ZZ, 02BH3ZZ, 02BH4ZZ, 025H0ZZ, 025H3ZZ, 025H4ZZ, 02TH0ZZ, 02TH3ZZ, 02TH4ZZ
CABG	ICD-9: 361, 3610, 3611, 3612, 3613, 3614, 3615, 3616, 3617, 3619 ICD-10: 0210093, 02100A3, 02100J3, 02100K3, 02100Z3, 0210493, 02104A3, 02104J3, 02104K3, 02104Z3, 021009W, 02100AW, 02100JW, 02100KW, 021049W, 02104AW, 02104JW, 02104KW, 021109W, 02110AW, 02110JW, 02110KW, 021149W, 02114AW, 02114JW, 02114KW, 021209W, 02120AW, 02120JW, 02120KW, 021249W, 02124AW, 02124JW, 02124KW, 021309W, 02130AW, 02130JW, 02130KW, 021349W, 02134AW, 02134JW, 02134KW, 0210098, 0210099, 021009C, 02100A8, 02100A9, 02100AC, 02100J8, 02100J9, 02100JC, 02100K8, 02100K9, 02100KC, 02100Z8, 02100Z9, 02100ZC, 0210498, 0210499, 021049C, 02104A8, 02104A9, 02104AC, 02104J8, 02104J9, 02104JC, 02104K8, 02104K9, 02104KC, 02104Z8, 02104Z9, 02104ZC, 0211098, 0211099, 021109C, 02110A8, 02110A9, 02110AC, 02110J8, 02110J9, 02110JC, 02110K8, 02110K9, 02110KC, 02110Z8, 02110Z9, 02110ZC, 0211498, 0211499, 021149C, 02114A8, 02114A9, 02114AC, 02114J8, 02114J9, 02114JC, 02114K8, 02114K9, 02114KC, 02114Z8, 02114Z9, 02114ZC, 021209C, 02120AC, 02120JC, 02120KC, 02120ZC, 021249C, 02124AC, 02124JC, 02124KC, 02124ZC, 021309C, 02130AC, 02130JC, 02130KC, 02130ZC, 021349C, 02134AC, 02134JC, 02134KC, 02134ZC, 021009F, 02100AF, 02100JF, 02100KF, 02100ZF, 021049F, 02104AF, 02104JF, 02104KF, 02104ZF, 0210093, 02100A3, 02100J3, 02100K3, 02100Z3, 0210493, 02104A3, 02104J3, 02104K3, 02104Z3, 02703ZZ, 02704ZZ, 02713ZZ, 02714ZZ, 02723ZZ, 02724ZZ, 02733ZZ, 02734ZZ, 02C03ZZ, 02700ZZ, 02710ZZ, 02720ZZ, 02730ZZ, 02C00ZZ, 02C10ZZ, 02C20ZZ, 02C30ZZ, 3E07017, 3E07317, 3E073PZ, 0211344, 0210444, 0213344, 0211444, 0271046, 0271346, 027134Z, 0272346, 027244Z, 0273346, 3E070PZ, 3E07317, 3E073PZ, 02C03ZZ, 02C04ZZ, 02C13ZZ, 02C14ZZ, 02C23ZZ, 02C24ZZ, 02C33ZZ, 02C34ZZ
Aortic surgery	ICD-9: 3804, 3805, 3814, 3815, 3834, 3835, 3845, 3865, 3885, 3973 ICD-10: 02QX0ZZ, 02QX3ZZ, 02QX4ZZ, 02RX07Z, 02RX0JZ, 02RX0KZ, 02RX47Z, 02RX4JZ, 02RX4KZ, 021X09A, 021X09B, 021X09D, 021X09P, 021X09Q, 021X09R, 021X0AA, 021X0AB, 021X0AD, 021X0AP, 021X0AQ, 021X0AR, 021X0JA, 021X0JB, 021X0JD, 021X0JP, 021X0JQ, 021X0JR, 021X0KA, 021X0KB, 021X0KD, 021X0KD, 021X0KP, 021X0KQ, 021X0KR
Mechanical circulatory assist device	ICD-9: 3741, 3760, 3765, 3766, 3768, 3762, 3965, 3966, 3761 ICD-10: 02HA0QZ, 02HA0RJ, 02HA0RS, 02HA0RZ, 02HA0YZ, 02HA3QZ, 02HA3RJ, 02HA3RS, 02HA3RZ, 02HA3YZ, 02HA4QZ, 02HA4RJ, 02HA4RS, 02HA4RZ, 02HA4YZ
PCI	ICD-9: 0066, 1755, 3601, 3602, 3603, 3604, 3605, 3606, 3607, 3608, 3609 ICD-10: 02703DZ, 02713EZ, 02723FZ, 02733GZ, 02703D6, 02713E6, 02723F6, 02733G6, 027034Z, 027135Z, 027236Z, 027337Z, 0270346, 0271356, 0272366, 0273376, 02703DZ, 02713EZ, 02723FZ, 02733GZ, 02703D6, 02713E6, 02723F6, 02733G6, 02C03ZZ, 02C13ZZ, 02C23ZZ, 02C33ZZ, 02C03Z6, 02C13Z6, 02C23Z6, 02C33Z6, 027034Z, 027135Z, 027236Z, 027337Z, 0270346, 0271356, 0272366, 0273376, 02C03ZZ, 02C13ZZ, 02C23ZZ, 02C33ZZ, 02C03Z6, 02C13Z6, 02C23Z6, 02C33Z6, 02703DZ, 02713EZ, 02723FZ, 02733GZ, 02703D6, 02713E6, 02723F6, 02733G6, 02C03ZZ, 02C13ZZ, 02C23ZZ, 02C33ZZ, 02C03Z6, 02C13Z6, 02C23Z6, 02C33Z6, 027034Z, 027135Z, 027236Z, 027337Z, 0270346, 0271356, 0272366, 0273376, 02C03ZZ, 02C13ZZ, 02C23ZZ, 02C33ZZ, 02C03Z6, 02C13Z6, 02C23Z6, 02C33Z6, 02703DZ, 02713EZ, 02723FZ, 02733GZ, 02703D6, 02713E6, 02723F6, 02733G6, 02C03ZZ, 02C13ZZ, 02C23ZZ, 02C33ZZ, 02C03Z6, 02C13Z6, 02C23Z6, 02C33Z6, 027034Z, 027135Z, 027236Z, 027337Z, 0270346, 0271356, 0272366, 0273376, 02C03ZZ, 02C13ZZ, 02C23ZZ, 02C33ZZ, 02C03Z6, 02C13Z6, 02C23Z6, 02C33Z6, ’02703DZ, 02713EZ, 02723FZ, 02733GZ, 02703D6, 02713E6, 02723F6, 02733G6, 02C03ZZ, 02C13ZZ, 02C23ZZ, 02C33ZZ, 02C03Z6,02C13Z6, 02C23Z6, 02C33Z6, 027034Z, 027135Z, 027236Z, 027337Z, 0270346, 0271356, 0272366, 0273376, 02C03ZZ, 02C13ZZ, 02C23ZZ, 02C33ZZ, 02C03Z6, 02C13Z6, 02C23Z6, 02C33Z6
Comorbidities	
Diabetes mellitus	ICD-9: 250.00, 250.01, 250.02, 250.03, 250.10, 250.11, 250.12, 250.13, 250.20, 250.21, 250.22, 250.23, 250.30, 250.31, 250.32, 250.33, 250.40, 250,41, 250.42, 250.43, 250.50, 250.51, 250.52. 250.53, 250.60, 250.61, 250.62, 250.63, 250.70, 250.71, 250.72, 250.73, 250.80, 250.81, 250.82, 250.83, 250.90, 250.91, 250.92, 250.93 ICD-10: E08.00, E08.01, E08.10, E08.11, E08.21, E08.22, E08.29, E08.311, E08.319, E08.321, E08.3211, E08.3212, E08.3213, E08.3219, E08.329, E08.3291, E08.3292, E08.3293, E08.3299, E08.331, E08.3311, E08.3312, E08.3313, E08.3319, E08.339, E08.3391, E08.3392, E08.3393, E08.3399, E08.341, E08.3411, E08.3412, E08.3413, E08.3419, E08.349, E08.3491, E08.3492, E08.3493, E08.3499, E08.351, E08.3511, E08.3512, E08.3513, E08.3519, E08.3521, E08.3522, E08.3523, E08.3529, E08.3531, E08.3532, E08.3533, E08.3539, E08.3541, E08.3542, E08.3543, E08.3549, E08.3551, E08.3552, E08.3553, E08.3559, E08.359, E08.3591, E08.3592, E08.3593, E08.3599, E08.36, E08.37X1, E08.37X2, E08.37X3, E08.37X9, E08.39, E08.40, E08.41, E08.42, E08.43, E08.44, E08.49, E08.51, E08.52, E08.59, E08.610, E08.618, E08.620, E08.621, E08.622, E08.628, E08.630, E08.638, E08.641, E08.649, E08.65, E08.69, E08.8, E08.9, E09.00, E09.01, E09.10, E09.11, E09.21, E09.22, E09.29, E09.311, E09.319, E09.321, E09.3211, E09.3212, E09.3213, E09.3219, E09.329, E09.3291, E09.3292, E09.3293, E09.3299, E09.331, E09.3311, E09.3312, E09.3313, E09.3319, E09.339, E09.3391, E09.3392, E09.3393, E09.3399, E09.341, E09.3411, E09.3412, E09.3413, E09.3419, E09.349, E09.3491, E09.3492, E09.3493, E09.3499, E09.351, E09.3511, E09.3512, E09.3513, E09.3519, E09.3521, E09.3522, E09.3523, E09.3529, E09.3531, E09.3532, E09.3533, E09.3539, E09.3541, E09.3542, E09.3543, E09.3549, E09.3551, E09.3552, E09.3553, E09.3559, E09.359, E09.3591, E09.3592, E09.3593, E09.3599, E09.36, E09.37X1, E09.37X2, E09.37X3, E09.37X9, E09.39, E09.40, E09.41, E09.42, E09.43, E09.44, E09.49, E09.51, E09.52, E09.59, E09.610, E09.618, E09.620, E09.621, E09.622, E09.628, E09.630, E09.638, E09.641, E09.649, E09.65, E09.69, E09.8, E09.9, E10.10, E10.11, E10.21, E10.22, E10.29, E10.311, E10.319, E10.321, E10.3211, E10.3212, E10.3213, E10.3219, E10.329, E10.3291, E10.3292, E10.3293, E10.3299, E10.331, E10.3311, E10.3312, E10.3313, E10.3319, E10.339, E10.3391, E10.3392, E10.3393, E10.3399, E10.341, E10.3411, E10.3412, E10.3413, E10.3419, E10.349, E10.3491, E10.3492, E10.3493, E10.3499, E10.351, E10.3511, E10.3512, E10.3513, E10.3519, E10.3521, E10.3522, E10.3523, E10.3529, E10.3531, E10.3532, E10.3533, E10.3539, E10.3541, E10.3542, E10.3543, E10.3549, E10.3551, E10.3552, E10.3553, E10.3559, E10.359, E10.3591, E10.3592, E10.3593, E10.3599, E10.36, E10.37X1, E10.37X2, E10.37X3, E10.37X9, E10.39, E10.40, E10.41, E10.42, E10.43, E10.44, E10.49, E10.51, E10.52, E10.59, E10.610, E10.618, E10.620, E10.621, E10.622, E10.628, E10.630, E10.638, E10.641, E10.649, E10.65, E10.69, E10.8, E10.9, E11.00, E11.01, E11.10, E11.11, E11.21, E11.22, E11.29, E11.311, E11.319, E11.321, E11.3211, E11.3212, E11.3213, E11.3219, E11.329, E11.3291, E11.3292, E11.3293, E11.3299, E11.331, E11.3311, E11.3312, E11.3313, E11.3319, E11.339, E11.3391, E11.3392, E11.3393, E11.3399, E11.341, E11.3411, E11.3412, E11.3413, E11.3419, E11.349, E11.3491, E11.3492, E11.3493, E11.3499, E11.351, E11.3511, E11.3512, E11.3513, E11.3519, E11.3521, E11.3522, E11.3523, E11.3529, E11.3531, E11.3532, E11.3533, E11.3539, E11.3541, E11.3542, E11.3543, E11.3549, E11.3551, E11.3552, E11.3553, E11.3559, E11.359, E11.3591, E11.3592, E11.3593, E11.3599, E11.36, E11.37X1, E11.37X2, E11.37X3, E11.37X9, E11.39, E11.40, E11.41, E11.42, E11.43, E11.44, E11.49, E11.51, E11.52, E11.59, E11.610, E11.618, E11.620, E11.621, E11.622, E11.628, E11.630, E11.638, E11.641, E11.649, E11.65, E11.69, E11.8, E11.9, E13.00, E13.01, E13.10, E13.11, E13.21, E13.22, E13.29, E13.311, E13.319, E13.321, E13.3211, E13.3212, E13.3213, E13.3219, E13.329, E13.3291, E13.3292, E13.3293, E13.3299, E13.331, E13.3311, E13.3312, E13.3313, E13.3319, E13.339, E13.3391, E13.3392, E13.3393, E13.3399, E13.341, E13.3411, E13.3412, E13.3413, E13.3419, E13.349, E13.3491, E13.3492, E13.3493, E13.3499, E13.351, E13.3511, E13.3512, E13.3513, E13.3519, E13.3521, E13.3522, E13.3523, E13.3529, E13.3531, E13.3532, E13.3533, E13.3539, E13.3541, E13.3542, E13.3543, E13.3549, E13.3551, E13.3552, E13.3553, E13.3559, E13.359, E13.3591, E13.3592, E13.3593, E13.3599, E13.36, E13.39, E13.40, E13.41, E13.42, E13.43, E13.44, E13.49, E13.51, E13.52, E13.59, E13.610, E13.618, E13.620, E13.621, E13.622, E13.628, E13.630, E13.638, E13.641, E13.649, E13.65, E13.69, E13.8, E13.9
CHF	ICD- 9: 428.0, 428.1, 428.20, 428.21, 428.22, 428.23, 428.30, 428.31, 428.32, 428.33, 428.40, 428.41, 428.42, 428.43, 428.9 ICD-10: I09.81, I11.0, I13.0, I13.2, I50.1, I50.20, I50.21, I50.22, I50.23, I50.30, I50.31, I50.32, I50.33, I50.40, I50.41, I50.42, I50.43, I50.810, I50.811, I50.812, I50.813, I50.814, I50.82, I50.83, I50.84, I50.89, I50.9
COPD	ICD-9: 490, 491.0, 491.1, 491.20, 491.21, 491.22, 491.8, 491.9, 492.0, 492.8, 496 ICD-10: J40, J41.0, J41.1, J41.8, J42, J43.0, J43.1, J43.2, J43.8, J43.9, J44.0, J44.1, J44.9, J47.0, J47.1, J47.9
Hypertension	ICD-9: 401.0, 401.1, 401.9, 402.00, 402.01, 403.10, 402.11, 402.90, 402.91, 403.00, 403.01, 403.10, 403.11, 403.90, 403.91, 404.00, 404.01, 404.02, 404.03, 404.10, 404.11, 404.12, 404.13, 404.90, 404.91, 404.92, 404.93, 405.01, 405.09, 405.11, 405.19, 405.91, 405.99 ICD-10: H35.031, H35.032, H35.033, H35.039, I10, I11.0, I11.9, I12.0, I12.9, I13.0, I13.10, I13.11, I13.2, I15.0, I15.1, I15.2, I15.8, I15.9, I67.4, N26.2
Hyperlipidemia	ICD-9: 272.0, 272.1, 272.2, 272.3, 272.4, 272.5, 272.6, 272.7, 272.8, 272.9 ICD-10: E78.0, E78.00, E78.01, E78.1, E78.2, E78.3, E78.4, E78.41, E78.49, E78.5
Atrial fibrillation	ICD-9: 427.3x ICD-10: I48.0, I48.1, I48.11, I48.19, I48.2, I48.20, I48.21, I48.91
CAD	ICD-9: 410.00, 410.01, 410.02, 410.10, 410.11, 410.12, 410.20, 410.21, 410.22, 410.30, 410.31, 410.32, 410.40, 410.41, 410.42, 410.50, 410.51, 410.52, 410.60, 410.61, 410.62, 410.70, 410.71, 410.72, 410.80, 410.81, 410.82, 410.90, 410.91, 410.92, 411.0, 411.1, 411.81, 411.89, 413.0, 413.1, 413.9, 414.00, 414.01, 414.02, 414.03, 414.04, 414.05, 414.06, 414.07, 414.10, 414.11, 414.12, 414.19, 414.2, 414.3, 414.4, 414.5, 414.6, 414.7, 414.8, 414.9 ICD-10: I20.0, I20.1, I20.8, I20.9, I21.01, I21.02, I21.09, I21.11, I21.19, I21.21, I21.29, I21.3, I21.4, I21.A1, I21.A9, I22.0, I22.1, I22.2, I22.8, I22.9, I23.0, I23.1, I23.2, I23.3, I23.4, I23.5, I23.6, I23.7, I23.8, I24.0, I24.1, I24.8, I24.9, I25.10, I25.110, I25.111, I25.118, I25.119, I25.3, I25.41, I25.42, I25.5, I25.6, I25.700, I25.701, I25.708, I25.709, I25.710, I25.711, I25.718, I25.719, I25.720, I25.721, I25.728, I25.729, I25.730, I25.731, I25.738, I25.739, I25.750, I25.751, I25.758, I25.759, I25.760, I25.761, I25.768, I25.769, I25.790, I25.791, I25.798, I25.799, I25.810, I25.811, I25.812, I25.82, I25.83, I25.84, I25.89, I25.9
PVD	ICD-9: 093.0, 437.3, 440.x, 441.x,443.1-443.9, 447.1, 557.1,557.9, V43.4 ICD-10: I70.x, I71.x, I73.1, I73.8, I73.9, I77.1,I79.0, I79.2, K55.1, K55.8, K55.9, Z95.8, Z95.9, 093.0, 437.3, 440.x, 441.x,443.1-443.9, 447.1, 557.1,557.9, V43.4
CVD	ICD-9: 434.00, 434.01, 434.10, 434.11, 434.90, 434.91, 435.0, 435.1, 435.2, 435.3, 435.8, 435.9 ICD-10: G45.0, G45.1, G45.2, G45.8, G45.9, G46.0, G46.1, G46.2, G46.3, G46.4, G46.5, G46.6, G46.7, G46.8, G97.31, G97.32, I60.00, I60.01, I60.02, I60.10, I60.11, I60.12, I60.20, I60.21, I60.22, I60.30, I60.31, I60.32, I60.4, I60.50, I60.51, I60.52, I60.6, I60.7, I60.8, I60.9, I61.0, I61.1, I61.2, I61.3, I61.4, I61.5, I61.6, I61.8, I61.9, I63.00, I63.011, I63.012, I63.013, I63.019, I63.02, I63.031, I63.032, I63.039, I63.09, I63.10, I63.111, I63.112, I63.113, I63.119, I63.12, I63.131, I63.132, I63.133, I63.139, I63.19, I63.20, I63.211, I63.212, I63.213, I63.219, I63.22, I63.231, I63.232, I63.233, I63.239, I63.29, I63.30, I63.311, I63.312, I63.313, I63.319, I63.321, I63.322, I63.323, I63.329, I63.331, I63.332, I63.333, I63.339, I63.341, I63.342, I63.343, I63.349, I63.39, I63.40, I63.411, I63.412, I63.413, I63.419, I63.421, I63.422, I63.423, I63.429, I63.431, I63.432, I63.433, I63.439, I63.441, I63.442, I63.443, I63.449, I63.49, I63.50, I63.511, I63.512, I63.513, I63.519, I63.521, I63.522, I63.523, I63.529, I63.531, I63.532, I63.533, I63.539, I63.541, I63.542, I63.543, I63.549, I63.59, I63.6, I63.8, I63.81, I63.89, I63.9, I66.01, I66.02, I66.03, I66.09, I66.11, I66.12, I66.13, I66.19, I66.21, I66.22, I66.23, I66.29, I66.3, I66.8, I66.9, I67.841, I67.848, I67.89, I97.810, I97.811, I97.820, I97.821
Previous MI	ICD-9: 412 ICD-10: I25.2
CKD	ICD-9: 585.1, 585.2, 585.3, 585.4, 585.5, 585.6, 585.9, 403.00, 403.01, 403.10, 403.11, 403.90, 403.91, 404.00, 404.01, 404.02, 404.03, 404.10, 404.11, 404.12, 404.13, 404.90, 404.91, 404.92, 404.93 ICD-10: A18.11, A52.75, B52.0, C64.1, C64.2, C64.9, C68.9, D30.00, D30.01, D30.02, D41.00, D41.01, D41.02, D41.10, D41.11, D41.12, D41.20, D41.21, D41.22, D59.3, E08.21, E08.22, E08.29, E08.65, E09.21, E09.22, E09.29, E10.21, E10.22, E10.29, E10.65, E11.21, E11.22, E11.29, E11.65, E13.21, E13.22, E13.29, E74.8, I12.0, I12.9, I13.0, I13.10, I13.11, I13.2, I70.1, I72.2, K76.7, M10.30, M10.311, M10.312, M10.319, M10.321, M10.322, M10.329, M10.331, M10.332, M10.339, M10.341, M10.342, M10.349, M10.351, M10.352, M10.359, M10.361, M10.362, M10.369, M10.371, M10.372, M10.379, M10.38, M10.39, M32.14, M32.15, M35.04, N00.0, N00.1, N00.2, N00.3, N00.4, N00.5, N00.6, N00.7, N00.8, N00.9, N00.A, N01.0, N01.1, N01.2, N01.3, N01.4, N01.5, N01.6, N01.7, N01.8, N01.9, N01.A, N02.0, N02.1, N02.2, N02.3, N02.4, N02.5, N02.6, N02.7, N02.8, N02.9, N02.A, N03.0, N03.1, N03.2, N03.3, N03.4, N03.5, N03.6, N03.7, N03.8, N03.9, N03.A, N04.0, N04.1, N04.2, N04.3, N04.4, N04.5, N04.6, N04.7, N04.8, N04.9, N04.A, N05.0, N05.1, N05.2, N05.3, N05.4, N05.5, N05.6, N05.7, N05.8, N05.9, N05.A, N06.0, N06.1, N06.2, N06.3, N06.4, N06.5, N06.6, N06.7, N06.8, N06.9, N06.A, N07.0, N07.1, N07.2, N07.3, N07.4, N07.5, N07.6, N07.7, N07.8, N07.9, N07.A, N08, N13.1, N13.2, N13.30, N13.39, N14.0, N14.1, N14.2, N14.3, N14.4, N15.0, N15.8, N15.9, N16, N17.0, N17.1, N17.2, N17.8, N17.9, N18.1, N18.2, N18.3, N18.30, N18.31, N18.32, N18.4, N18.5, N18.6, N18.9, N19, N25.0, N25.1, N25.81, N25.89, N25.9, N26.1, N26.9, Q61.02, Q61.11, Q61.19, Q61.2, Q61.3, Q61.4, Q61.5, Q61.8, Q62.0, Q62.2, Q62.10, Q62.11, Q62.12, Q62.31, Q62.32, Q62.39, R94.4
Concomitant procedures	
Tricuspid valve repair	ICD-9: 3514 ICD-10: 02QJ0ZG, 02QJ0ZZ, 02QJ3ZG, 02QJ3ZZ, 02QJ4ZG, 02QJ4ZZ
Surgical ablation	ICD-9: 3733, 3734 ICD-10: 0256, 0257, 0258
LAAO	ICD-9: 3736 ICD-10: 02L7, 02B7, 0257
In-hospital outcomes	
Acute pulmonary insufficiency following thoracic surgery	ICD-9: 518.51 ICD-10: J95.1
Acute renal failure	ICD-9: 584.x ICD-10: N99.0
Blood transfusion	ICD-9: 9900, 9901, 9902, 9903, 9904 ICD-10: 30233N1, 30243N1, 30253N1, 30233H0, 30243H0, 30253H0
PPMI	ICD-9: 3780, 3871, 3782, 3783, 3785, 3786, 3787, 3794, 3795, 3796, 3797, 3798 ICD-10: 0JH604Z, 0JH605Z, 0JH606Z, 0JH607Z, 0JH608Z, OJH609Z
CVA following cardiac surgery	ICD-9: 997.02 ICD-10: I97.810, I97.820
Reintervention	
Mitral valve reintervention	ICD-9: 3502, 3512, 3523, 3524, 3597 ICD-10: 02QG0ZE, 02QG0ZZ, 02QG3ZE, 02QG3ZZ, 02QG4ZE, 02QG4ZZ, 02RG07Z, 02RG08Z, 02RG0JZ, 02RG0KZ, 02VG0ZZ, 02CG0ZZ, 02WG07Z, 02WG08Z, 02WG0JZ, 02WG0KZ, 02RG37H, 02RG37Z, 02RG38H, 02RG38Z, 02RG3JH, 02RG3JZ, 02RG3KH, 02RG3KZ, 02UG3JZ

*ICD-9*, International Classification of Diseases, Ninth Revision; *ICD-10*, International Classification of Diseases, Tenth Revision; *MVr*, mitral valve repair; *CABG*, coronary artery bypass grafting; *PCI*, percutaneous coronary intervention; *CHF*, congestive heart failure; *CAD*, coronary artery disease; *COPD*, chronic obstructive pulmonary disease; *PVD*, peripheral vascular disease; *CVD*, cerebrovascular disease; *MI*, myocardial infarction; *CKD*, chronic kidney disease; *LAAO*, left atrial appendage occlusion; *PPMI*, permanent pacemaker implantation; *CVA*, cerebrovascular accident.

**Table E2:** Postoperative morbidity and mortality following MVr in unmatched groups

Parameter	Robotic MVr group (N = 1502)	Conventional MVr group (N = 12,182)	*P* value
Hospital length of stay, d, mean ± SD	6.3 ± 3.8	8.8 ± 5.9	<.001
Acute pulmonary insufficiency, n (%)	73 (4.9)	676 (5.6)	.27
Acute renal failure, n (%)	117 (7.8)	1681 (14)	<.001
Blood product transfusion, n (%)	261 (17)	2826 (23)	<.001
PPMI, n (%)	29 (1.9)	603 (5)	<.001
CVA, n (%)	12 (0.8)	78 (0.64)	.47
Mitral valve reintervention, n (%)	69 (4.6)	546 (4.5)	.90
Mortality, n (%)			
30-d	14 (0.93)	220 (1.8)	.013
1-y	36 (2.4)	644 (5.3)	<.001
5-y	125 (8.3)	1852 (15)	<.001

*MVr*, Mitral valve repair; *SD*, standard deviation; *PPMI*, permanent pacemaker implantation; *CVA*, cerebrovascular accident.

**Table E3:** Occurrence of PPMI following robotic MVr with and without tricuspid valve repair in propensity score–matched groups

Parameter	Robotic MVr with tricuspid valve repair (N = 38)	Robotic MVr without tricuspid valve repair (N = 1087)	*P* value
PPMI, n (%)	0 (0)	23 (2)	.37

*PPMI*, Permanent pacemaker implantation; *MVr*, mitral valve repair.
