# Supplementary material for: Long-term mortality following robotic mitral valve repair
Source: JTCVS Struct Endovasc. Author manuscript; Available in PMC 2026 Apr 8. (PMC13056376; doi:10.1016/j.xjse.2025.100090)
Supplement: Supplementary file 1 [file NIHMS2161973-supplement-1.pdf]

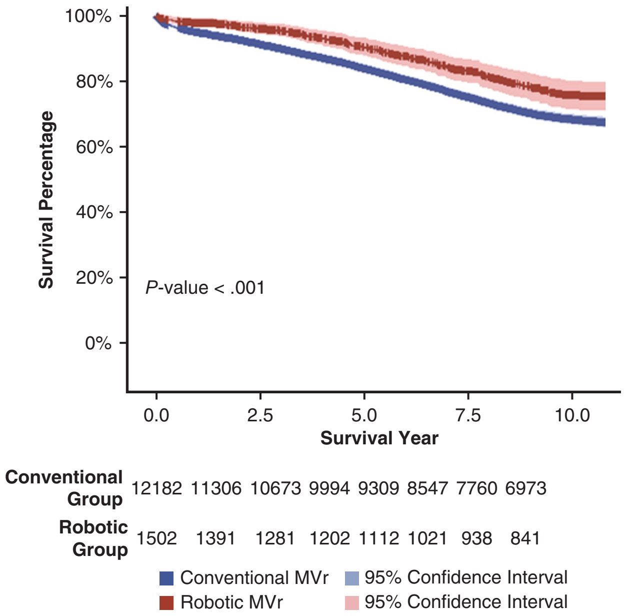

FIGURE E1. Unadjusted survival curves following conventional and robotic mitral valve repair (*MVr*).

TABLE E1. ICD-9 and ICD-10 codes used for data collection

Inclusion criteria	
Surgical MVr	ICD-9: 3502, 3512
	ICD-10: 02QG0ZE, 02QG0ZZ, 02QG3ZE, 02QG3ZZ, 02QG4ZE, 02QG4ZZ
Robotic system used	ICD-9: 17.41, 17.42, 17.43, 17.44, 17.45, 17.49
	ICD-10: 8E0W0CZ, 8E0W0EN, 8E0W0EZ, 8E0W3CZ, 8E0W3EN, 8E0W3EZ, 8E0W4CZ, 8E0W4EN, 8E0W4EZ, 8E0W7CZ, 8E0W7EN, 8E0W7EZ, 8E0W8CZ, 8E0W8EN, 8E0W8EZ, 8E0WXBF, 8E0WXBG, 8E0WXBH, 8E0WXBZ, 8E0WXCZ, 8E0WXY8
Exclusion criteria	
Rheumatic mitral valve disease or mitral valve stenosis	ICD-9: 394, 3940, 3941, 3942, 3949, 396, 3960, 3961, 3962, 3963, 3968, 3969
	ICD-10: I080, I081, I083, I088, I089, I050, I051, I059, I052, I058, I342
Endocarditis	ICD-9: 421, 4210, 4211, 4219, 4249, 42490, 42491, 42499, 11281, 07422, 4148, 4149
	ICD-10: I011, A3282, A3951, A5203, A5483, B3321, B376, I33, I330, I339, I38, I39, M3211
Congenital cardiac disease	ICD-9: 7450, 7451, 74510, 74511, 74512, 74519, 7452, 7453, 7454, 74560, 74561, 74569, 7457, 7458, 7462, 7465, 7466, 7467
	ICD-10: Q210, Q221, Q231, Q244, Q2543, Q263, Q87418, Q20, Q200, Q201, Q202, Q203, Q204, Q205, Q206, Q208, Q209, Q213, Q212, Q218, Q219, Q225, Q232, Q233, Q234, Q225
Cardiomyopathy	ICD-9: 425, 4250, 4251, 42511, 42518, 4252, 4253, 4254, 4255, 4257, 4258, 4259
	ICD-10: I255, I421, I420, I422, I427, I428, I429, I42, I421, I423, I424, I425, I426, I43
Myocardial infarction	ICD-9: 410, 4100, 41001, 41002, 41003, 4101, 41010, 41011, 41012, 4102, 41020, 41021, 41022, 4103, 41030, 41031, 41032, 4104, 41040, 41041, 41042, 4105, 41050, 41051, 41052, 4106, 41060, 41061, 410, 4100, 41001, 41002, 41003, 4101, 41010, 41011, 41012, 4102, 41020, 41021, 41022, 4103, 41030, 41031, 41032, 4104, 41040, 41041, 41042, 4105, 41050, 41051, 41052, 4106, 41060, 41061, 41062, 4107, 41070, 41071, 41072, 4108, 41080, 41081, 41081, 4109, 41090, 41091, 41092, 41062, 4107, 41070, 41071, 41072, 4108, 41080, 41081, 41081, 4109, 41090, 41091, 41092
	ICD-10: All I21, I22, I23 codes (I210, I2101, I2102, I211, I2111, I2119, I212, I2121, I2129, I213, I214, I219, I213, I213, I214, I21A, I21A1, I219), I252, all I257
Prior CABG	ICD-9: V45.81
	ICD-10: Z95.1
Prior valve replacement	ICD-9: V43.3, V42.2
	ICD-10: Z95.2, Z95.3, Z95.4
Prior heart assist device	ICD-9: V43.21
	ICD-10: Z95.811
Prior heart transplant	ICD-9: 3751
	ICD-10: Z94.1
Aortic valve surgery	ICD-9: 3521, 3522, 3511, 3501, 3505, 3506, 3509, 3510, 3511, 3533, 3595, 3596, 3599
	ICD-10: 02RF07Z, 02RF08Z, 02RF0JZ, 02RF0KZ, 02RF37H, 02RF37Z, 02RF38H, 02RF38Z, 02RF3JH, 02RF3JZ, 02RF3KH, 02RF3KZ, 02RF47Z, 02RF48Z, 02RF4JZ, 02RF4KZ, 02QF0ZZ, 02QF3ZZ, 02QF4ZZ, 027F0ZZ, 027F3ZZ, 027F4ZZ, 027F04Z, 027F34Z, 027F44Z, 027F0DZ, 027F3DZ, 027F4DZ, 02NF0ZZ, 02NF3ZZ, 02NF4ZZ, 02UF07Z, 02UF0JZ, 02UF0KZ, 02UF37Z, 02UF3JZ, 02UF3KZ, 02UF47Z, 02UF4JZ, 02UF4KZ, 02BF0ZZ, 02BF3ZZ, 02BF4ZZ, 025F0ZZ, 025F3ZZ, 025F4ZZ, X2RF032, X2RF432, X2RF332, 027F34Z, 027F44Z, 02QF0ZJ, 02QF3ZJ, 02QF4ZJ, 02WF07Z, 02WF08Z, 02WF0JZ, 02WF0KZ, 02WF37Z, 02WF38Z, 02WF3JZ, 02WF3KZ, 02WF47Z, 02WF48Z, 02WF4JZ, 02WF4KZ, 025F0ZZ, 025F3ZZ, 025F4ZZ, 027F04Z, 027F0DZ, 027F0ZZ, 027F3DZ, 027F3ZZ, 027F4DZ, 027F4ZZ, 02BF0ZZ, 02BF3ZZ, 02BF4ZZ, 02CF0ZZ, 02CF3ZZ, 02CF4ZZ, 02NF0ZZ, 02NF3ZZ, 02NF4ZZ, 02QF0ZZ, 02QF3ZZ, 02QF4ZZ
Pulmonic valve surgery	ICD-9: 3525, 3526, 3513, 3503, 3507, 3508
	ICD-10: 02RH07Z, 02RH08Z, 02RH0JZ, 02RH0KZ, 02RH37H, 02RH37Z, 02RH38H, 02RH38Z, 02RH3JH, 02RH3JZ, 02RH3KH, 02RH3KZ, 02RH47Z, 02RH48Z, 02RH4JZ, 02RH4KZ, 02QH0ZZ, 02QH3ZZ, 02QH4ZZ, 027H0ZZ, 027H3ZZ, 027H4ZZ, 027H04Z, 027H34Z, 027H44Z, 027H0DZ, 027H3DZ, 027H4DZ, 02NH0ZZ, 02NH3ZZ, 02NH4ZZ, 02UH07Z, 02UH0JZ, 02UH0KZ, 02UH37Z, 02UH3JZ, 02UH3KZ, 02UH47Z, 02UH4JZ, 02UH4KZ, 02BH0ZZ, 02BH3ZZ, 02BH4ZZ, 025H0ZZ, 025H3ZZ, 025H4ZZ, 02TH0ZZ, 02TH3ZZ, 02TH4ZZ
CABG	ICD-9: 361, 3610, 3611, 3612, 3613, 3614, 3615, 3616, 3617, 3619
	ICD-10: 0210093, 02100A3, 02100J3, 02100K3, 02100Z3, 0210493, 02104A3, 02104J3, 02104K3, 02104Z3, 021009W, 02100AW, 02100JW, 02100KW, 021049W, 02104AW, 02104JW, 02104KW, 021109W, 02110AW, 02110JW, 02110KW, 021149W, 02114AW, 02114JW, 02114KW, 021209W, 02120AW,

(Continued)

TABLE E1. Continued

	02120JW, 02120KW, 021249W, 02124AW, 02124JW, 02124KW, 021309W, 02130AW, 02130JW, 02130KW, 021349W, 02134AW, 02134JW, 02134KW, 0210098, 0210099, 021009C, 02100A8, 02100A9, 02100AC, 02100J8, 02100J9, 02100JC, 02100K8, 02100K9, 02100KC, 02100Z8, 02100Z9, 02100ZC, 0210498, 0210499, 021049C, 02104A8, 02104A9, 02104AC, 02104J8, 02104J9, 02104JC, 02104K8, 02104K9, 02104KC, 02104Z8, 02104Z9, 02104ZC, 0211098, 0211099, 021109C, 02110A8, 02110A9, 02110AC, 02110J8, 02110J9, 02110JC, 02110K8, 02110K9, 02110KC, 02110Z8, 02110Z9, 02110ZC, 0211498, 0211499, 021149C, 02114A8, 02114A9, 02114AC, 02114J8, 02114J9, 02114JC, 02114K8, 02114K9, 02114KC, 02114Z8, 02114Z9, 02114ZC, 021209C, 02120AC, 02120JC, 02120KC, 02120ZC, 021249C, 02124AC, 02124JC, 02124KC, 02124ZC, 021309C, 02130AC, 02130JC, 02130KC, 02130ZC, 021349C, 02134AC, 02134JC, 02134KC, 02134ZC, 021009F, 02100AF, 02100JF, 02100KF, 02100ZF, 021049F, 02104AF, 02104JF, 02104KF, 02104ZF, 0210093, 02100A3, 02100J3, 02100K3, 02100Z3, 0210493, 02104A3, 02104J3, 02104K3, 02104Z3, 02703ZZ, 02704ZZ, 02713ZZ, 02714ZZ, 02723ZZ, 02724ZZ, 02733ZZ, 02734ZZ, 02C03ZZ, 02700ZZ, 02710ZZ, 02720ZZ, 02730ZZ, 02C00ZZ, 02C10ZZ, 02C20ZZ, 02C30ZZ, 3E07017, 3E07317, 3E073PZ, 0211344, 0210444, 0213344, 0211444, 0271046, 0271346, 027134Z, 0272346, 027244Z, 0273346, 3E070PZ, 3E07317, 3E073PZ, 02C03ZZ, 02C04ZZ, 02C13ZZ, 02C14ZZ, 02C23ZZ, 02C24ZZ, 02C33ZZ, 02C34ZZ
Aortic surgery	ICD-9: 3804, 3805, 3814, 3815, 3834, 3835, 3845, 3865, 3885, 3973
	ICD-10: 02QX0ZZ, 02QX3ZZ, 02QX4ZZ, 02RX07Z, 02RX0JZ, 02RX0KZ, 02RX47Z, 02RX4JZ, 02RX4KZ, 021X09A, 021X09B, 021X09D, 021X09P, 021X09Q, 021X09R, 021X0AA, 021X0AB, 021X0AD, 021X0AP, 021X0AQ, 021X0AR, 021X0JA, 021X0JB, 021X0JD, 021X0JP, 021X0JQ, 021X0JR, 021X0KA, 021X0KB, 021X0KD, 021X0KD, 021X0KP, 021X0KQ, 021X0KR
Mechanical circulatory assist device	ICD-9: 3741, 3760, 3765, 3766, 3768, 3762, 3965, 3966, 3761
	ICD-10: 02HA0QZ, 02HA0RJ, 02HA0RS, 02HA0RZ, 02HA0YZ, 02HA3QZ, 02HA3RJ, 02HA3RS, 02HA3RZ, 02HA3YZ, 02HA4QZ, 02HA4RJ, 02HA4RS, 02HA4RZ, 02HA4YZ
PCI	ICD-9: 0066, 1755, 3601, 3602, 3603, 3604, 3605, 3606, 3607, 3608, 3609
	ICD-10: 02703DZ, 02713EZ, 02723FZ, 02733GZ, 02703D6, 02713E6, 02723F6, 02733G6, 027034Z, 027135Z, 027236Z, 027337Z, 0270346, 0271356, 0272366, 0273376, 02703DZ, 02713EZ, 02723FZ, 02733GZ, 02703D6, 02713E6, 02723F6, 02733G6, 02C03ZZ, 02C13ZZ, 02C23ZZ, 02C33ZZ, 02C03Z6, 02C13Z6, 02C23Z6, 02C33Z6, 027034Z, 027135Z, 027236Z, 027337Z, 0270346, 0271356, 0272366, 0273376, 02C03ZZ, 02C13ZZ, 02C23ZZ, 02C33ZZ, 02C03Z6, 02C13Z6, 02C23Z6, 02C33Z6, 02703DZ, 02713EZ, 02723FZ, 02733GZ, 02703D6, 02713E6, 02723F6, 02733G6, 02C03ZZ, 02C13ZZ, 02C23ZZ, 02C33ZZ, 02C03Z6, 02C13Z6, 02C23Z6, 02C33Z6, 027034Z, 027135Z, 027236Z, 027337Z, 0270346, 0271356, 0272366, 0273376, 02C03ZZ, 02C13ZZ, 02C23ZZ, 02C33ZZ, 02C03Z6, 02C13Z6, 02C23Z6, 02C33Z6, 02703DZ, 02713EZ, 02723FZ, 02733GZ, 02703D6, 02713E6, 02723F6, 02733G6, 02C03ZZ, 02C13ZZ, 02C23ZZ, 02C33ZZ, 02C03Z6, 02C13Z6, 02C23Z6, 02C33Z6, 027034Z, 027135Z, 027236Z, 027337Z, 0270346, 0271356, 0272366, 0273376, 02C03ZZ, 02C13ZZ, 02C23ZZ, 02C33ZZ, 02C03Z6, 02C13Z6, 02C23Z6, 02C33Z6, '02703DZ, 02713EZ, 02723FZ, 02733GZ, 02703D6, 02713E6, 02723F6, 02733G6, 02C03ZZ, 02C13ZZ, 02C23ZZ, 02C33ZZ, 02C03Z6,02C13Z6, 02C23Z6, 02C33Z6, 027034Z, 027135Z, 027236Z, 027337Z, 0270346, 0271356, 0272366, 0273376, 02C03ZZ, 02C13ZZ, 02C23ZZ, 02C33ZZ, 02C03Z6, 02C13Z6, 02C23Z6, 02C33Z6
Comorbidities	
Diabetes mellitus	ICD-9: 250.00, 250.01, 250.02, 250.03, 250.10, 250.11, 250.12, 250.13, 250.20, 250.21, 250.22, 250.23, 250.30, 250.31, 250.32, 250.33, 250.40, 250,41, 250.42, 250.43, 250.50, 250.51, 250.52. 250.53, 250.60, 250.61, 250.62, 250.63, 250.70, 250.71, 250.72, 250.73, 250.80, 250.81, 250.82, 250.83, 250.90, 250.91, 250.92, 250.93
	ICD-10: E08.00, E08.01, E08.10, E08.11, E08.21, E08.22, E08.29, E08.311, E08.319, E08.321, E08.3211, E08.3212, E08.3213, E08.3219, E08.329, E08.3291, E08.3292, E08.3293, E08.3299, E08.331, E08.3311, E08.3312, E08.3313, E08.3319, E08.339, E08.3391, E08.3392, E08.3393, E08.3399, E08.341, E08.3411, E08.3412, E08.3413, E08.3419, E08.349, E08.3491, E08.3492, E08.3493, E08.3499, E08.351, E08.3511, E08.3512, E08.3513, E08.3519, E08.3521, E08.3522, E08.3523, E08.3529, E08.3531, E08.3532, E08.3533, E08.3539, E08.3541, E08.3542, E08.3543, E08.3549, E08.3551, E08.3552, E08.3553, E08.3559, E08.359, E08.3591, E08.3592, E08.3593, E08.3599, E08.36, E08.37X1, E08.37X2, E08.37X3, E08.37X9, E08.39, E08.40, E08.41, E08.42, E08.43, E08.44, E08.49, E08.51, E08.52, E08.59, E08.610, E08.618, E08.620, E08.621, E08.622, E08.628, E08.630, E08.638, E08.641, E08.649, E08.65, E08.69, E08.8, E08.9, E09.00, E09.01, E09.10, E09.11, E09.21, E09.22, E09.29, E09.311, E09.319, E09.321, E09.3211, E09.3212, E09.3213, E09.3219, E09.329, E09.3291, E09.3292, E09.3293, E09.3299, E09.331,

(Continued)

TABLE E1. Continued

	E09.3311, E09.3312, E09.3313, E09.3319, E09.339, E09.3391, E09.3392, E09.3393, E09.3399, E09.341, E09.3411, E09.3412, E09.3413, E09.3419, E09.349, E09.3491, E09.3492, E09.3493, E09.3499, E09.351, E09.3511, E09.3512, E09.3513, E09.3519, E09.3521, E09.3522, E09.3523, E09.3529, E09.3531, E09.3532, E09.3533, E09.3539, E09.3541, E09.3542, E09.3543, E09.3549, E09.3551, E09.3552, E09.3553, E09.3559, E09.359, E09.3591, E09.3592, E09.3593, E09.3599, E09.36, E09.37X1, E09.37X2, E09.37X3, E09.37X9, E09.39, E09.40, E09.41, E09.42, E09.43, E09.44, E09.49, E09.51, E09.52, E09.59, E09.610, E09.618, E09.620, E09.621, E09.622, E09.628, E09.630, E09.638, E09.641, E09.649, E09.65, E09.69, E09.8, E09.9, E10.10, E10.11, E10.21, E10.22, E10.29, E10.311, E10.319, E10.321, E10.3211, E10.3212, E10.3213, E10.3219, E10.329, E10.3291, E10.3292, E10.3293, E10.3299, E10.331, E10.3311, E10.3312, E10.3313, E10.3319, E10.339, E10.3391, E10.3392, E10.3393, E10.3399, E10.341, E10.3411, E10.3412, E10.3413, E10.3419, E10.349, E10.3491, E10.3492, E10.3493, E10.3499, E10.351, E10.3511, E10.3512, E10.3513, E10.3519, E10.3521, E10.3522, E10.3523, E10.3529, E10.3531, E10.3532, E10.3533, E10.3539, E10.3541, E10.3542, E10.3543, E10.3549, E10.3551, E10.3552, E10.3553, E10.3559, E10.359, E10.3591, E10.3592, E10.3593, E10.3599, E10.36, E10.37X1, E10.37X2, E10.37X3, E10.37X9, E10.39, E10.40, E10.41, E10.42, E10.43, E10.44, E10.49, E10.51, E10.52, E10.59, E10.610, E10.618, E10.620, E10.621, E10.622, E10.628, E10.630, E10.638, E10.641, E10.649, E10.65, E10.69, E10.8, E10.9, E11.00, E11.01, E11.10, E11.11, E11.21, E11.22, E11.29, E11.311, E11.319, E11.321, E11.3211, E11.3212, E11.3213, E11.3219, E11.329, E11.3291, E11.3292, E11.3293, E11.3299, E11.331, E11.3311, E11.3312, E11.3313, E11.3319, E11.339, E11.3391, E11.3392, E11.3393, E11.3399, E11.341, E11.3411, E11.3412, E11.3413, E11.3419, E11.349, E11.3491, E11.3492, E11.3493, E11.3499, E11.351, E11.3511, E11.3512, E11.3513, E11.3519, E11.3521, E11.3522, E11.3523, E11.3529, E11.3531, E11.3532, E11.3533, E11.3539, E11.3541, E11.3542, E11.3543, E11.3549, E11.3551, E11.3552, E11.3553, E11.3559, E11.359, E11.3591, E11.3592, E11.3593, E11.3599, E11.36, E11.37X1, E11.37X2, E11.37X3, E11.37X9, E11.39, E11.40, E11.41, E11.42, E11.43, E11.44, E11.49, E11.51, E11.52, E11.59, E11.610, E11.618, E11.620, E11.621, E11.622, E11.628, E11.630, E11.638, E11.641, E11.649, E11.65, E11.69, E11.8, E11.9, E13.00, E13.01, E13.10, E13.11, E13.21, E13.22, E13.29, E13.311, E13.319, E13.321, E13.3211, E13.3212, E13.3213, E13.3219, E13.329, E13.3291, E13.3292, E13.3293, E13.3299, E13.331, E13.3311, E13.3312, E13.3313, E13.3319, E13.339, E13.3391, E13.3392, E13.3393, E13.3399, E13.341, E13.3411, E13.3412, E13.3413, E13.3419, E13.349, E13.3491, E13.3492, E13.3493, E13.3499, E13.351, E13.3511, E13.3512, E13.3513, E13.3519, E13.3521, E13.3522, E13.3523, E13.3529, E13.3531, E13.3532, E13.3533, E13.3539, E13.3541, E13.3542, E13.3543, E13.3549, E13.3551, E13.3552, E13.3553, E13.3559, E13.359, E13.3591, E13.3592, E13.3593, E13.3599, E13.36, E13.39, E13.40, E13.41, E13.42, E13.43, E13.44, E13.49, E13.51, E13.52, E13.59, E13.610, E13.618, E13.620, E13.621, E13.622, E13.628, E13.630, E13.638, E13.641, E13.649, E13.65, E13.69, E13.8, E13.9
CHF	ICD- 9: 428.0, 428.1, 428.20, 428.21, 428.22, 428.23, 428.30, 428.31, 428.32, 428.33, 428.40, 428.41, 428.42, 428.43, 428.9
	ICD-10: I09.81, I11.0, I13.0, I13.2, I50.1, I50.20, I50.21, I50.22, I50.23, I50.30, I50.31, I50.32, I50.33, I50.40, I50.41, I50.42, I50.43, I50.810, I50.811, I50.812, I50.813, I50.814, I50.82, I50.83, I50.84, I50.89, I50.9
COPD	ICD-9: 490, 491.0, 491.1, 491.20, 491.21, 491.22, 491.8, 491.9, 492.0, 492.8, 496
	ICD-10: J40, J41.0, J41.1, J41.8, J42, J43.0, J43.1, J43.2, J43.8, J43.9, J44.0, J44.1, J44.9, J47.0, J47.1, J47.9
Hypertension	ICD-9: 401.0, 401.1, 401.9, 402.00, 402.01, 403.10, 402.11, 402.90, 402.91, 403.00, 403.01, 403.10, 403.11, 403.90, 403.91, 404.00, 404.01, 404.02, 404.03, 404.10, 404.11, 404.12, 404.13, 404.90, 404.91, 404.92, 404.93, 405.01, 405.09, 405.11, 405.19, 405.91, 405.99
	ICD-10: H35.031, H35.032, H35.033, H35.039, I10, I11.0, I11.9, I12.0, I12.9, I13.0, I13.10, I13.11, I13.2, I15.0, I15.1, I15.2, I15.8, I15.9, I67.4, N26.2
Hyperlipidemia	ICD-9: 272.0, 272.1, 272.2, 272.3, 272.4, 272.5, 272.6, 272.7, 272.8, 272.9
	ICD-10: E78.0, E78.00, E78.01, E78.1, E78.2, E78.3, E78.4, E78.41, E78.49, E78.5
Atrial fibrillation	ICD-9: 427.3x
	ICD-10: I48.0, I48.1, I48.11, I48.19, I48.2, I48.20, I48.21, I48.91
CAD	ICD-9: 410.00, 410.01, 410.02, 410.10, 410.11, 410.12, 410.20, 410.21, 410.22, 410.30, 410.31, 410.32, 410.40, 410.41, 410.42, 410.50, 410.51, 410.52, 410.60, 410.61, 410.62, 410.70, 410.71, 410.72, 410.80, 410.81, 410.82, 410.90, 410.91, 410.92, 411.0, 411.1, 411.81, 411.89, 413.0, 413.1, 413.9, 414.00, 414.01, 414.02, 414.03, 414.04, 414.05, 414.06, 414.07, 414.10, 414.11, 414.12, 414.19, 414.2, 414.3, 414.4, 414.5, 414.6, 414.7, 414.8, 414.9

(Continued)

TABLE E1. Continued

	ICD-10: I20.0, I20.1, I20.8, I20.9, I21.01, I21.02, I21.09, I21.11, I21.19, I21.21, I21.29, I21.3, I21.4, I21.A1, I21.A9, I22.0, I22.1, I22.2, I22.8, I22.9, I23.0, I23.1, I23.2, I23.3, I23.4, I23.5, I23.6, I23.7, I23.8, I24.0, I24.1, I24.8, I24.9, I25.10, I25.110, I25.111, I25.118, I25.119, I25.3, I25.41, I25.42, I25.5, I25.6, I25.700, I25.701, I25.708, I25.709, I25.710, I25.711, I25.718, I25.719, I25.720, I25.721, I25.728, I25.729, I25.730, I25.731, I25.738, I25.739, I25.750, I25.751, I25.758, I25.759, I25.760, I25.761, I25.768, I25.769, I25.790, I25.791, I25.798, I25.799, I25.810, I25.811, I25.812, I25.82, I25.83, I25.84, I25.89, I25.9
PVD	ICD-9: 093.0, 437.3, 440.x, 441.x,443.1-443.9, 447.1, 557.1,557.9, V43.4
	ICD-10: I70.x, I71.x, I73.1, I73.8, I73.9, I77.1,I79.0, I79.2, K55.1, K55.8, K55.9, Z95.8, Z95.9, 093.0, 437.3, 440.x, 441.x,443.1-443.9, 447.1, 557.1,557.9, V43.4
CVD	ICD-9: 434.00, 434.01, 434.10, 434.11, 434.90, 434.91, 435.0, 435.1, 435.2, 435.3, 435.8, 435.9
	ICD-10: G45.0, G45.1, G45.2, G45.8, G45.9, G46.0, G46.1, G46.2, G46.3, G46.4, G46.5, G46.6, G46.7, G46.8, G97.31, G97.32, I60.00, I60.01, I60.02, I60.10, I60.11, I60.12, I60.20, I60.21, I60.22, I60.30, I60.31, I60.32, I60.4, I60.50, I60.51, I60.52, I60.6, I60.7, I60.8, I60.9, I61.0, I61.1, I61.2, I61.3, I61.4, I61.5, I61.6, I61.8, I61.9, I63.00, I63.011, I63.012, I63.013, I63.019, I63.02, I63.031, I63.032, I63.039, I63.09, I63.10, I63.111, I63.112, I63.113, I63.119, I63.12, I63.131, I63.132, I63.133, I63.139, I63.19, I63.20, I63.211, I63.212, I63.213, I63.219, I63.22, I63.231, I63.232, I63.233, I63.239, I63.29, I63.30, I63.311, I63.312, I63.313, I63.319, I63.321, I63.322, I63.323, I63.329, I63.331, I63.332, I63.333, I63.339, I63.341, I63.342, I63.343, I63.349, I63.39, I63.40, I63.411, I63.412, I63.413, I63.419, I63.421, I63.422, I63.423, I63.429, I63.431, I63.432, I63.433, I63.439, I63.441, I63.442, I63.443, I63.449, I63.49, I63.50, I63.511, I63.512, I63.513, I63.519, I63.521, I63.522, I63.523, I63.529, I63.531, I63.532, I63.533, I63.539, I63.541, I63.542, I63.543, I63.549, I63.59, I63.6, I63.8, I63.81, I63.89, I63.9, I66.01, I66.02, I66.03, I66.09, I66.11, I66.12, I66.13, I66.19, I66.21, I66.22, I66.23, I66.29, I66.3, I66.8, I66.9, I67.841, I67.848, I67.89, I97.810, I97.811, I97.820, I97.821
Previous MI	ICD-9: 412
	ICD-10: I25.2
CKD	ICD-9: 585.1, 585.2, 585.3, 585.4, 585.5, 585.6, 585.9, 403.00, 403.01, 403.10, 403.11, 403.90, 403.91, 404.00, 404.01, 404.02, 404.03, 404.10, 404.11, 404.12, 404.13, 404.90, 404.91, 404.92, 404.93
	ICD-10: A18.11, A52.75, B52.0, C64.1, C64.2, C64.9, C68.9, D30.00, D30.01, D30.02, D41.00, D41.01, D41.02, D41.10, D41.11, D41.12, D41.20, D41.21, D41.22, D59.3, E08.21, E08.22, E08.29, E08.65, E09.21, E09.22, E09.29, E10.21, E10.22, E10.29, E10.65, E11.21, E11.22, E11.29, E11.65, E13.21, E13.22, E13.29, E74.8, I12.0, I12.9, I13.0, I13.10, I13.11, I13.2, I70.1, I72.2, K76.7, M10.30, M10.311, M10.312, M10.319, M10.321, M10.322, M10.329, M10.331, M10.332, M10.339, M10.341, M10.342, M10.349, M10.351, M10.352, M10.359, M10.361, M10.362, M10.369, M10.371, M10.372, M10.379, M10.38, M10.39, M32.14, M32.15, M35.04, N00.0, N00.1, N00.2, N00.3, N00.4, N00.5, N00.6, N00.7, N00.8, N00.9, N00.A, N01.0, N01.1, N01.2, N01.3, N01.4, N01.5, N01.6, N01.7, N01.8, N01.9, N01.A, N02.0, N02.1, N02.2, N02.3, N02.4, N02.5, N02.6, N02.7, N02.8, N02.9, N02.A, N03.0, N03.1, N03.2, N03.3, N03.4, N03.5, N03.6, N03.7, N03.8, N03.9, N03.A, N04.0, N04.1, N04.2, N04.3, N04.4, N04.5, N04.6, N04.7, N04.8, N04.9, N04.A, N05.0, N05.1, N05.2, N05.3, N05.4, N05.5, N05.6, N05.7, N05.8, N05.9, N05.A, N06.0, N06.1, N06.2, N06.3, N06.4, N06.5, N06.6, N06.7, N06.8, N06.9, N06.A, N07.0, N07.1, N07.2, N07.3, N07.4, N07.5, N07.6, N07.7, N07.8, N07.9, N07.A, N08, N13.1, N13.2, N13.30, N13.39, N14.0, N14.1, N14.2, N14.3, N14.4, N15.0, N15.8, N15.9, N16, N17.0, N17.1, N17.2, N17.8, N17.9, N18.1, N18.2, N18.3, N18.30, N18.31, N18.32, N18.4, N18.5, N18.6, N18.9, N19, N25.0, N25.1, N25.81, N25.89, N25.9, N26.1, N26.9, Q61.02, Q61.11, Q61.19, Q61.2, Q61.3, Q61.4, Q61.5, Q61.8, Q62.0, Q62.2, Q62.10, Q62.11, Q62.12, Q62.31, Q62.32, Q62.39, R94.4
Concomitant procedures	
Tricuspid valve repair	ICD-9: 3514
	ICD-10: 02QJ0ZG, 02QJ0ZZ, 02QJ3ZG, 02QJ3ZZ, 02QJ4ZG, 02QJ4ZZ
Surgical ablation	ICD-9: 3733, 3734
	ICD-10: 0256, 0257, 0258
LAAO	ICD-9: 3736
	ICD-10: 02L7, 02B7, 0257
In-hospital outcomes	
Acute pulmonary insufficiency following thoracic surgery	ICD-9: 518.51
	ICD-10: J95.1
Acute renal failure	ICD-9: 584.x
	ICD-10: N99.0

(Continued)

TABLE E1. Continued

Blood transfusion	ICD-9: 9900, 9901, 9902, 9903, 9904
	ICD-10: 30233N1, 30243N1, 30253N1, 30233H0, 30243H0, 30253H0
PPMI	ICD-9: 3780, 3871, 3782, 3783, 3785, 3786, 3787, 3794, 3795, 3796, 3797, 3798
	ICD-10: 0JH604Z, 0JH605Z, 0JH606Z, 0JH607Z, 0JH608Z, OJH609Z
CVA following cardiac surgery	ICD-9: 997.02
	ICD-10: I97.810, I97.820
Reintervention	
Mitral valve reintervention	ICD-9: 3502, 3512, 3523, 3524, 3597
	ICD-10: 02QG0ZE, 02QG0ZZ, 02QG3ZE, 02QG3ZZ, 02QG4ZE, 02QG4ZZ, 02RG07Z, 02RG08Z, 02RG0JZ, 02RG0KZ, 02VG0ZZ, 02CG0ZZ, 02WG07Z, 02WG08Z, 02WG0JZ, 02WG0KZ, 02RG37H, 02RG37Z, 02RG38H, 02RG38Z, 02RG3JH, 02RG3JZ, 02RG3KH, 02RG3KZ, 02UG3JZ

ICD-9, International Classification of Diseases, Ninth Revision; *ICD-10*, International Classification of Diseases, Tenth Revision; *MVr*, mitral valve repair; *CABG*, coronary artery bypass grafting; *PCI*, percutaneous coronary intervention; *CHF*, congestive heart failure; *CAD*, coronary artery disease; *COPD*, chronic obstructive pulmonary disease; *PVD*, peripheral vascular disease; *CVD*, cerebrovascular disease; *MI*, myocardial infarction; *CKD*, chronic kidney disease; *LAAO*, left atrial appendage occlusion; *PPMI*, permanent pacemaker implantation; *CVA*, cerebrovascular accident.

TABLE E2. Postoperative morbidity and mortality following MVr in unmatched groups

Parameter	Robotic MVr group (N = 1502)	Conventional MVr group (N = 12,182)	*P* value
Hospital length of stay, d, mean ± SD	6.3 ± 3.8	8.8 ± 5.9	<.001
Acute pulmonary insufficiency, n (%)	73 (4.9)	676 (5.6)	.27
Acute renal failure, n (%)	117 (7.8)	1681 (14)	<.001
Blood product transfusion, n (%)	261 (17)	2826 (23)	<.001
PPMI, n (%)	29 (1.9)	603 (5)	<.001
CVA, n (%)	12 (0.8)	78 (0.64)	.47
Mitral valve reintervention, n (%)	69 (4.6)	546 (4.5)	.90
Mortality, n (%)			
30-d	14 (0.93)	220 (1.8)	.013
1-y	36 (2.4)	644 (5.3)	<.001
5-y	125 (8.3)	1852 (15)	<.001

MVr, Mitral valve repair; *SD*, standard deviation; *PPMI*, permanent pacemaker implantation; *CVA*, cerebrovascular accident.

TABLE E3. Occurrence of PPMI following robotic MVr with and without tricuspid valve repair in propensity score–matched groups

Parameter	Robotic MVr with tricuspid valve repair (N = 38)	Robotic MVr without tricuspid valve repair (N = 1087)	*P* value
PPMI, n (%)	0 (0)	23 (2)	.37

PPMI, Permanent pacemaker implantation; *MVr*, mitral valve repair.
